# FGFR3-driven gene regulatory network analysis reveals a protumoral role for p63 in luminal bladder tumors

**DOI:** 10.1172/JCI193280

**Published:** 2026-08-03

**Authors:** Aura Moreno-Vega, Macarena Zambrano, Lilia Estrada-Virrueta, Xiangyu Meng, Julia Puig, Helene Neyret-Kahn, Mingjun Shi, Florent Dufour, Guerric Gilbert, Ke Li, Clarice Groeneveld, Jacqueline Fontugne, Mercedes Pérez-Escavy, Wajdi Dhifli, Clément Hua, Luc Cabel, Clémentine Krucker, Laura Tanguy, Sia Viborg Lindskrog, Claire Beraud, Yanina V. Langle, Tao Ye, Fariza Tahi, Irwin Davidson, Jesus M. Paramio, Lars Dyrskjøt, Yves Allory, Philippe Lluel, Ana Maria Eiján, Mohamed Elati, François Radvanyi, Catalina Lodillinsky, Isabelle Bernard-Pierrot

**Affiliations:** 1 Institut Curie, Centre National de Recherche Scientifique (CNRS), UMR144, Molecular Oncology team, PSL Research University, Sorbonne Université UPMC Université Paris 6, Paris, France.; 2 Universidad de Buenos Aires, Facultad de Medicina, Instituto de Oncología Ángel H. Roffo, Área de Investigación, Buenos Aires, Argentina.; 3 Université de Paris-Saclay, Université d’Evry, IBISC, Evry, France.; 4 Health Science Center, Hubei Minzu University, Enshi, China.; 5 Univ. Lille, CNRS, INSERM, CHU Lille, UMR9020 – UMR-1277 - Canther, F-59000 Lille, France.; 6 Department of Urology, Beijing Friendship Hospital, Capital Medical University, Beijing, China.; 7 Institut Curie, CNRS UMR9029, INSERM U1353, IMPACT team, Paris, France.; 8 Department of Pathology, Institut Curie, Saint-Cloud, France.; 9 Université Versailles St-Quentin, Université Paris-Saclay, F-78180, Montigny-le-Bretonneux, France.; 10 Molecular and Translational Oncology Division, Centro de Investigaciones Energéticas, Medioambientales y Tecnológicas (CIEMAT); Cell and Molecular Oncology, Instituto de Investigación Hospital “12 de Octubre”; and CIBER Cancer (CIBERONC), Madrid, Spain.; 11 Department of Molecular Medicine, Aarhus University Hospital, Aarhus, Denmark.; 12 Department of Clinical Medicine, Aarhus University, Aarhus, Denmark.; 13 Urosphere, Toulouse, France.; 14 Institut de Génétique et de Biologie Moléculaire et Cellulaire (IGBMC), INSERM U1258, CNRS UMR7104, Université de Strasbourg, Illkirch, France.; 15 Consejo Nacional de Investigaciones Científicas y Técnicas (CONICET), Buenos Aires, Argentina.

**Keywords:** Genetics, Oncology, Molecular biology, Oncogenes, Urology

## Abstract

Fibroblast growth factor receptor 3 (*FGFR3*) is one of the most frequently altered genes in bladder cancer, primarily through activating mutations that drive oncogenesis and are enriched in luminal tumors. However, the underlying gene regulatory network (GRN) remains poorly characterized. Here, we constructed an *FGFR3*-mutated GRN using a bottom-up bioinformatics approach, integrating transcriptomic data from bladder cancer cell lines, *FGFR3*-mutated tumors, and *FGFR3* perturbation experiments in human and mouse models. Using publicly available CRISPR/Cas9 screening data, we identified transcription factors from this GRN that regulate the viability of *FGFR3*-mutated cells, with a focus on p63 (*TP63*). We showed that FGFR3 activation upregulates p63 in patient-derived xenografts and cell lines, while single-cell RNA sequencing revealed heterogeneous p63 activation associated with basal differentiation. Functional studies, including *TP63* knockdown in FGFR3-dependent in vitro and in vivo models and RNA-seq along with p63 ChIP-seq, demonstrated that p63 directly promotes cell proliferation and migration and uncovered a positive feedback loop between FGFR3 and p63. Together, these findings support p63 as a protumorigenic regulator in *FGFR3-*mutated tumors despite their luminal differentiation and provide a detailed FGFR3-driven GRN, offering insights into FGFR3-induced oncogenic dependency and potential strategies to circumvent resistance to FGFR inhibitors.

## Abstract 2

FGFR3 mutations drive bladder carcinogenesis. This study identifies a FGFR3 network, including p63, revealing oncogenic dependencies and offering insights into tumor biology and therapies

## Introduction

Bladder cancer (BLCa) is the fourth most common cancer in men in industrialized countries and can be divided into 2 main groups based on tumor stages. Non-muscle-invasive bladder carcinoma (NMIBC) is the most frequent form at first diagnosis, accounting for 75% of patients. Although NMIBC has a favorable prognosis with an 80% 5-year survival rate, a substantial proportion of patients (70%) experience recurrence after initial treatment. Moreover, depending on tumor grade and stage, 5%–75% of NMIBCs progress to muscle-invasive bladder cancer (MIBC) (1). Unlike NMIBC, MIBC is a life-threatening disease with a 5-year survival of less than 60%, which drops below 6% in the presence of metastasis (2). Activating mutations in fibroblast growth factor receptor 3 (*FGFR3*) are among the most frequent genetic alterations in BLCa, occurring in more than 65% of NMIBCs (enriched in class 1 and class 3 subtypes) and 15% of MIBCs (enriched in the luminal papillary [LumP] subtype) (3–5). Moreover, translocations leading to *FGFR3* gene fusions can be observed in 3% of MIBCs (6). Recently, promising results have been reported in clinical trials targeting FGFR3 (7–9), leading to the FDA approval of the first pan-FGFRs in advanced BLCa. However, as observed in preclinical BLCa models and with other targeted therapies in various cancer types (EGFR, BRAF, KIT; in lung cancer, melanoma, gastrointestinal stromal tumors, respectively), patients develop resistance to RTK-targeting treatment (8). In 30% of cases, secondary mutations at *FGFR3* gatekeeper residues have been reported, and nongenetic resistance mechanisms have also been observed, such as EGFR overexpression (10).

This observation led to the proposal of a combined treatment with anti-FGFR and anti-EGFR agents to overcome resistance (10, 11). Since the FGFR3 gene regulatory network (GRN) in BLCa remains poorly characterized, gaining a deeper understanding of this network should allow us to better comprehend the role of FGFR3 in the disease and uncover new therapeutic targets, such as master regulators, to prevent resistance observed with anti-FGFR treatments (12). Identifying MYC, a key transcription factor (TF) activated by FGFR3, has allowed us to propose potential therapeutic strategies by inhibiting the FGFR3-MYC regulatory loop (9).

Various bioinformatics methods exist to infer GRNs from high-throughput data, enabling the discovery of critical pathways and core regulatory circuitry involving master regulators. We used the hybrid-learning cooperative regulatory network (H-LICORN) algorithm, which integrates data mining methods with numerical linear regression, to efficiently infer a context-specific GRN (13, 14). Specifically, we predicted a cooperative network of transcription factors and cofactors (TFs/coTFs; coactivators and corepressors) using transcriptomic data from *FGFR3*-mutated BLCa cell lines and human bladder tumors. Experimentally derived data, in which the expression or activity of mutant FGFR3 was modulated in both in vitro and in vivo preclinical models, enabled us to highlight the TFs and coTFs driven by FGFR3 in the network. Then, we identified essential regulators of this network using publicly available cell viability data from a large CRISPR/Cas9-based screen conducted across 5 FGFR3-dependent BLCa cell lines (15). Among them, we identified p63 (encoded by *TP63*). We confirmed that FGFR3 activation regulated p63 expression in both *FGFR3*-mutated patient-derived xenografts (PDXs) and cell lines. However, single-cell RNA-seq analysis revealed heterogeneous p63 activation in *FGFR3*-mutated LumP PDXs and cell lines, correlating with the expression of basal differentiation markers. *TP63* knockdown demonstrated that, in addition to its established role in basal cells, p63 promotes tumor growth, cell proliferation, and migration in *FGFR3*-mutated BLCa cell lines. By integrating transcriptomic data following *TP63* knockdown with p63 ChIP-seq analyses in BLCa cell lines, we identified direct p63-regulated transcriptional programs involved in these processes, which show strong similarities to those described in basal contexts. Our analyses also uncovered a positive feedback loop between FGFR3 and p63.

This study, by identifying key master regulators within FGFR3-induced GRN, including p63, provides deeper insight into the biology of *FGFR3*-mutated tumors and may offer potential therapeutic opportunities to circumvent resistance to FGFR inhibitors observed in clinical settings.

## Results

### BLCa GRN of TFs and coTFs in FGFR3-mutated tumors.

**Figure 1:**
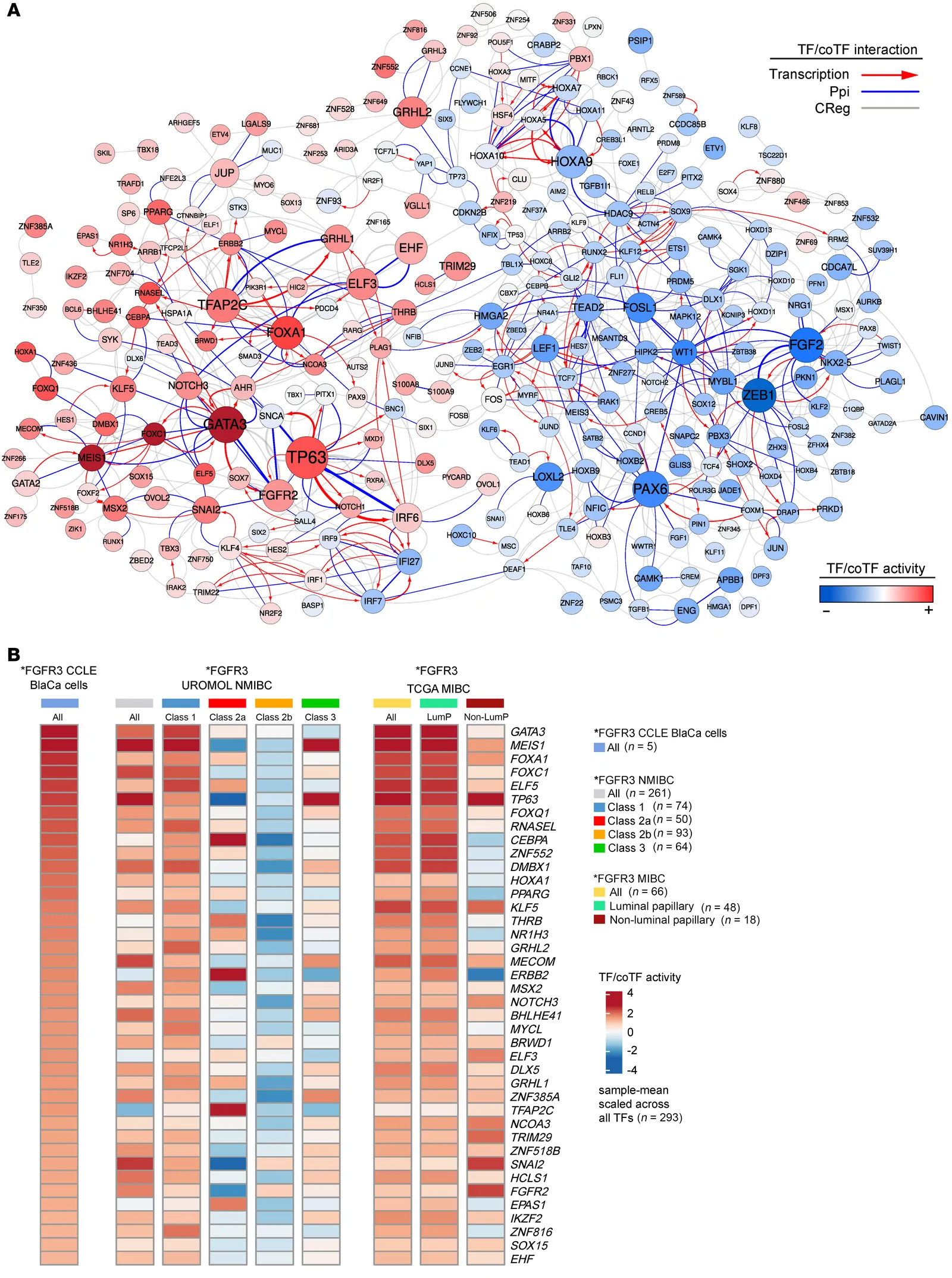
Transcriptional coregulatory network in *FGFR3*-altered bladder cancer cells and tumors. (**A**) The cooperativity network inferred from 36 bladder cancer–derived cell lines (BLCa-GRN) transcriptomes and TFs’ activity calculated only in *FGFR3*-altered cell lines (*FGFR3, *n* = 5) (Supplemental Table 2). Nodes represent transcription factors and cofactors (TFs/coTFs), with coregulatory interactions shown in gray (H-LICORN algorithm), blue (published protein-protein interactions; PPIs), and red arrows (transcriptional regulation). Node color (red = high, blue = low) indicates TF/coTF activity in *FGFR3 cells, with node size proportional to the number of targets and color intensity reflecting activation level. (**B**) Heatmap showing the 40 most active TFs/coTFs in *FGFR3 BLCa cell lines and *FGFR3 tumors across NMIBC (*n* = 261; UROMOL, UROMOL, a European multicenter cohort of patients with NMIBC) and MIBC (*n* = 66; TCGA) and across molecular subtypes.

To minimize bias associated with heterogeneous transcriptomic data from bladder tumors, particularly due to stromal contamination, we reconstructed the GRN using transcriptomic data from the 36 BLCa-derived cell lines included in the Cancer Cell Line Encyclopedia (CCLE) 2019Q1 database (see Methods). We applied the CoRegNet package, which implements the H-LICORN algorithm, to infer the GRN and further refined it by integrating protein-protein interactions (PPIs) and transcriptional regulatory interactions (transcription factor–binding sites; TFBS) (see Methods). The resulting GRN comprised 720 TFs/coTFs, 6,374 target genes, and 31,003 regulatory interactions, which were significantly enriched for validated PPIs (*P* value = 6.34 × 10^–127^) and TFBS (*P* value < 1 × 10^–100^). Based on shared targets between pairs of TFs/coTFs, the GRN was transformed into a cooperativity network (referred to as the coregulatory BLCa-GRN) (Figure 1A).

To identify the transcriptional programs active in an *FGFR3*-mutated context, we assessed the activity of each TF/coTF from the expression of their target genes using the CoRegNet algorithm (Supplemental Table 1; supplemental material available online with this article; https://doi.org/10.1172/JCI193280DS1) and CCLE transcriptomic data from 5 FGFR3-dependent BLCa cell lines expressing FGFR3 mutants (fusion proteins or activating point mutations): RT112 (FGFR3-TACC3), RT112-84 (FGFR3-TACC3), RT4 (FGFR3-TACC3), SW-780 (FGFR3-BAIAP2L1), and UM-UC-14 (FGFR3-S249C) (Supplemental Table 2). The computed activity scores were then mapped onto the inferred BLCa-GRN (Figure 1A).

We also calculated the activity of the TFs/coTFs using transcriptomic data from 2 independent cohorts of *FGFR3*-mutated tumors: 261 NMIBCs from UROMOL (4) and 66 MIBCs from The Cancer Genome Atlas (TCGA) (6) (Supplemental Table 2). We focused on the 40 most active TFs/coTFs in the FGFR3-dependent BLCa cell lines and found that they were also active in the *FGFR3*-mutated NMIBC and MIBC tumors (36/40 and 40/40, respectively) (Figure 1B). However, when stratifying tumors by molecular subtype, these same TFs were consistently active in *FGFR3*-mutated LumP MIBCs and class 1 NMIBCs (40/40), whereas other subtypes, particularly classes 2a and 2b, displayed more divergent activation patterns.

These differences are likely because the analyzed cell lines are mainly derived from MIBC and classified as LumP (16, 17) and the luminal-like differentiation of class 1 NMIBCs (4, 5). Supporting the biological relevance of our reconstructed network, we identified several previously described BLCa driver genes, including master regulators known to drive luminal cell identity in LumP tumors. These included GATA3, PPARG, FOXA1, KLF5, TRIM29, and NOTCH3 (Figure 1B) (15, 17–21).

Our results highlighted both subtype-specific and shared transcriptional programs within the *FGFR3*-mutated tumors, with common TFs/coTFs across *FGFR3*-mutated subtypes likely representing core regulators of the FGFR3 oncogenic signaling pathway (Figure 1B). TF activity patterns were highly similar across the 5 FGFR3-dependent cell lines from CCLE and the MGH-U3 cell line (FGFR3-Y375C), regardless of whether *FGFR3* alterations were translocations leading to expression of fusion proteins or point mutations (Supplemental Figure 1A), though mutated cell lines clustered together based on cosine similarity (Supplemental Figure 1B).

### Dependency on FGFR3-driven TFs in FGFR3-mutated BLCa cells, with a focus on p63.

Among the TFs activated in *FGFR3*-mutated tumors and cell lines (Supplemental Table 2), we sought to identify those directly regulated by FGFR3. We took advantage of published transcriptomic data obtained after inhibition of FGFR3 with the pan-FGFR inhibitor AZD4547 for 24 hours in 2 FGFR3-dependent human BLCa cell lines (22), RT112 and MGH-U3. We also used our previously published transcriptomic data from urothelial hyperplasia and bladder tumors of mice overexpressing FGFR3-S249C, together with normal urothelium from littermate controls (23). In this model, the expression of human FGFR3IIIb carrying the S249C mutation was specifically targeted to the urothelium using the murine uroplakin II promoter, leading to the development of hyperplastic lesions and low-grade papillary tumors from 6 and 18 months of age, respectively.

**Figure 2:**
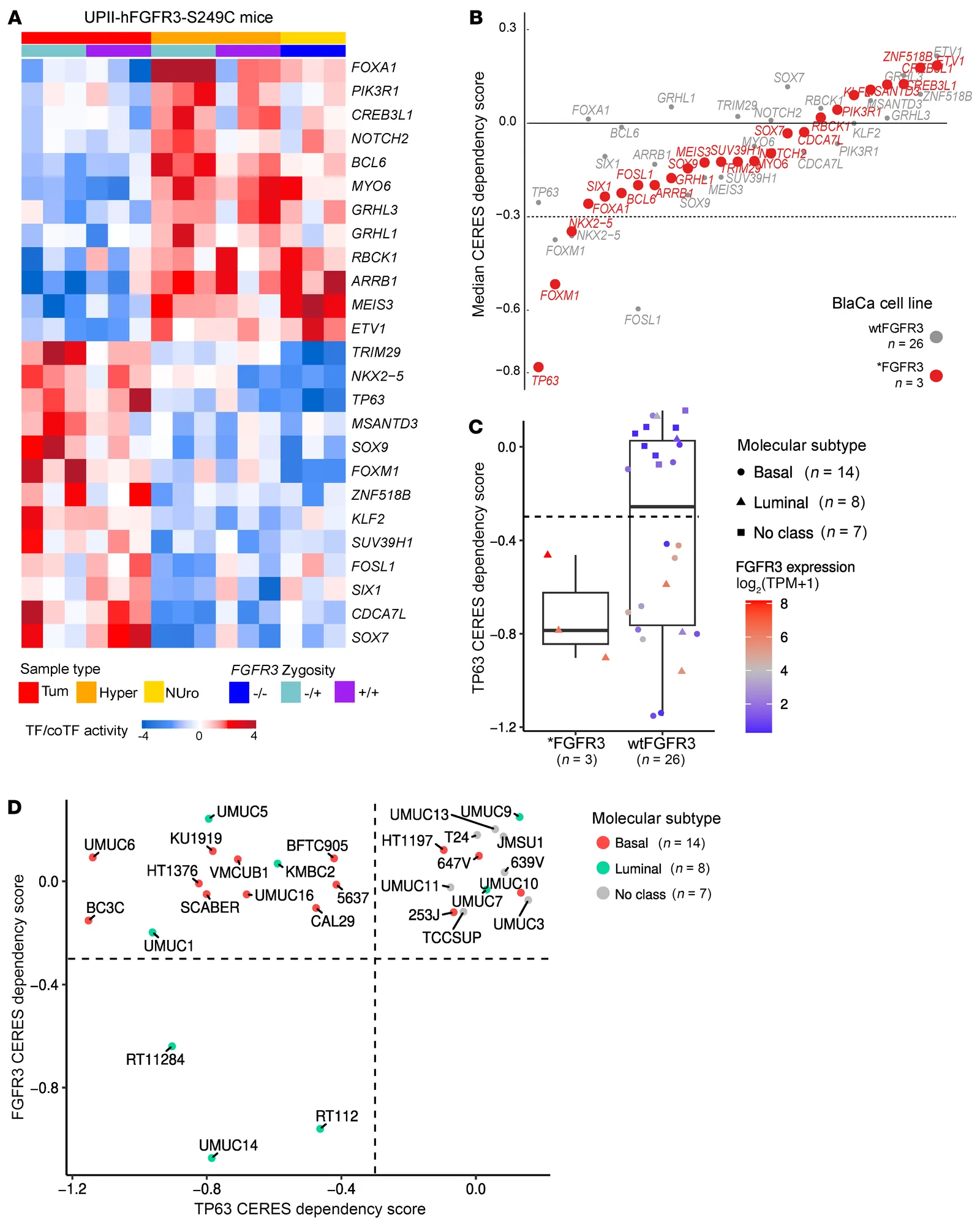
Identification of FGFR3-regulated TFs and discovery of *TP63* as a key viability gene. (**A**) Heatmap showing the activity of 25 FGFR3-regulated TFs/coTFs identified by integrating transcriptomic data from FGFR3 inhibition in bladder cancer cell lines and FGFR3-S249C–induced mouse bladder tumors (Supplemental Figure 1C). TF activity in mouse samples: normal urothelium, hyperplasic urothelium, and bladder tumors. (**B**) Impact of CRISPR/Cas9 KO (DepMap, CERES scores) of the 25 FGFR3-regulated TFs on cell viability in FGFR3-dependent (*FGFR3, red) versus non-FGFR3-dependent bladder cancer cell lines. (**C** and **D**) Effect of CRISPR/Cas9 KO of *TP63* on cell viability in bladder cancer cell lines according to *FGFR3* status (**C**) and FGFR3 dependency (**D**). Box plots show the interquartile range, median (line), and minimum and maximum (whiskers). Molecular subtypes were determined using our previously established single cell–derived classifier (16, 17). TPM, transcripts per million.

Cross-species analysis of transcriptomic data from human and mouse *FGFR3*-mutated tumors showed highly concordant transcriptomic programs (23), supporting the relevance of this model to decipher the FGFR3-driven signaling pathway. We assessed TF/coTF activity in each dataset and identified TFs with significantly altered activities (either up- or downregulated) after FGFR3 inhibition in cell lines or in FGFR3-induced mouse tumors compared with normal urothelium. This analysis identified 25 regulators displaying opposite activation patterns upon FGFR3 inhibition in BLCa cell lines or FGFR3-S249C overexpression in mouse bladder tumors (Supplemental Figure 1C). Notably, compared with normal urothelium, the TFs’ and coTFs’ activities (13 activated and 12 repressed by FGFR3) were not changed in FGFR3-S249C–induced hyperplasia but were specifically altered in tumors (Figure 2A), suggesting a role in malignant cell transformation.

To further assess the functional relevance of these regulators, we analyzed publicly available data from genome-wide gene dependency screening (Broad Institute, AVANA CRISPR-Cas9 dataset 2019Q3, https://depmap.org/portal/download/all/?release=DepMap+Public+19Q3). We calculated median CERES (Computational Estimation of gene dependencies from large-scale RNAi and CRISPR Essentiality Screens) dependency scores for each of these 25 genes in 3 FGFR3-dependent cell lines expressing FGFR3 mutants and compared them with scores from 26 BLCa cell lines expressing wild-type FGFR3 (Figure 2B). Only *TP63*, *FOXM1*, and *NKX2-5* were identified as important for viability of FGFR3-dependent cells, based on a CERES dependency score below –0.3 as the threshold. Notably, the knockout of *TP63* (which encodes p63) had the strongest effect on the viability of FGFR3-dependent BLCa cells. This effect was even more pronounced than that in wild-type *FGFR3* cells (Figure 2, B and C). However, consistent with the well-established role of p63 in squamous/basal tumors in general and basal MIBC in particular (24–26), basal BLCa cell lines were *TP63* dependent in this screen (Figure 2, C and D), and 3 wild-type*FGFR3* luminal cell lines were also *TP63* dependent (Figure 2C) but independent of *FGFR3* expression for their viability (Figure 2D). Given this unexpected link between FGFR3 activation, classically associated with luminal differentiation and p63 activation/dependency, we focused our study on deciphering the regulatory connection between FGFR3 and p63 and elucidating the functional role of p63 in *FGFR3*-mutated tumors.

### p63 activation is associated with FGFR3 pathway activity and basal-like cell states within luminal tumors.

Although p63 emerged as a highly active TF in *FGFR3*-mutated tumors in both MIBC and NMIBC cohorts (Figure 1B), FGFR3 activation mechanisms are not restricted to these genetic alterations. To further characterize the relationship between FGFR3 signaling and p63 activation, and to determine whether *FGFR3* mutation status or FGFR3 pathway activity is associated with p63 activation, we analyzed p63 activity relative to FGFR3 signaling across UROMOL and TCGA cohorts using our previously established FGFR3 pathway signature derived from siRNA-mediated FGFR3 knockdown in FGFR3-dependent cell lines (23).

**Figure 3:**
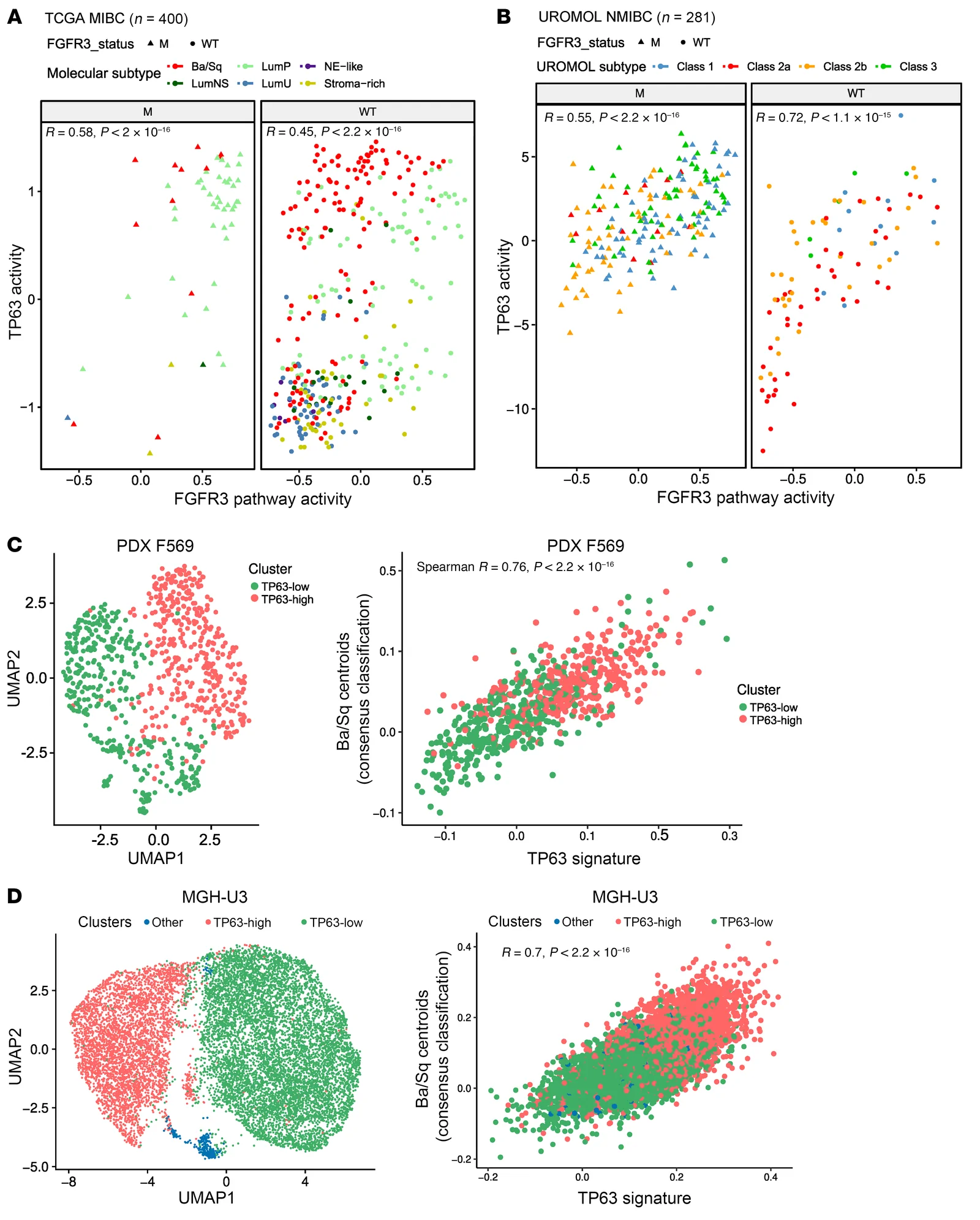
p63 activation correlates with FGFR3 pathway activity and basal-like cell states within luminal bladder tumors. (**A** and **B**) Correlation between TP63 GRN-regulon activity and FGFR3 pathway activation signature in individual MIBC tumors from TCGA cohort (**A**) and individual NMIBC tumors from the UROMOL cohort (Supplemental Table 2) (**B**), stratified by *FGFR3* mutation status and molecular subtype. (**C** and **D**) Single-cell RNA-seq analysis of tumor cells from an *FGFR3*-S249C PDX (F659) (**C**) and of MGH-U3 cells (**D**). Uniform manifold approximation and projection (UMAP) representation showing clusters with high or low *TP63* expression (left). Correlation between TP63 activity and basal/squamous cell differentiation scores (right). (**A**–**D**) Correlation analyses were performed using Spearman’s rank correlation coefficient.

In MIBC, p63 regulon activity positively correlated with FGFR3 pathway activation, independently of *FGFR3* mutation status (Figure 3A). LumP tumors showed the highest concordance between the FGFR3 pathway and p63 activity, whereas basal/squamous tumors presented elevated p63 activity with low FGFR3 pathway activation, consistent with lineage-specific p63 regulation. Similar results were observed in NMIBC, where p63 activity correlates with FGFR3 pathway activation in both *FGFR3*-mutated and wild-type FGFR3 tumors (Figure 3B). Class 1 and 3 NMIBC showed strong FGFR3–p63 concordance, while class 2 tumors displayed greater heterogeneity.

To determine whether this variability reflects intratumoral heterogeneity within FGFR3-driven luminal tumors, we analyzed single-cell RNA-seq data from an FGFR3-S249C PDX (F659) (Figure 3C) and MGH-U3 cells (Figure 3D). In both models, unsupervised clustering identified 2 tumor cell populations defined by high or low TP63 mRNA expression and p63 regulon activity. FGFR3 pathway activation was observed across clusters (Supplemental Figure 2, A and B). Integration of p63 activity scores with basal lineage-specific transcriptional signature revealed that p63-high cells exhibited increased basal/squamous differentiation compared with p63-low cells, despite arising within an overall luminal FGFR3-driven tumor context. p63 activity correlated with basal score (Figure 3, C and D) but not with proliferation (Supplemental Figure 2, C and D), indicating that heterogeneity primarily reflects differences in differentiation state rather than cell cycle. Consistently, despite their distinct p63 activity scores, both MGH-U3 cell clusters exhibited a comparable antiproliferative response to FGFR treatment, indicating that baseline p63 activity does not predict sensitivity to FGFR3-dependent growth inhibition (Supplemental Figure 2C).

Despite heterogeneous p63 activation in *FGFR3*-mutated tumors and its association with basal-like cell states — a feature generally linked to poor prognosis in BLCa — p63 activity did not stratify clinical outcomes among *FGFR3*-mutated tumors. Specifically, no differences in overall survival were observed between TP63-high and TP63-low *FGFR3*-mutated tumors in TCGA MIBC cohort (Supplemental Figure 3A). Similarly, p63 activity did not predict progression-free or recurrence-free survival in *FGFR3*-mutated NMIBCs from the UROMOL cohort (Supplemental Figure 3, B and C).

Together, these data demonstrated that FGFR3 pathway activation is associated with heterogeneous p63 activation and the emergence of basal-like cell states within luminal tumors. These observations led us to investigate the molecular mechanisms by which FGFR3 regulates p63 activity and p63 function in FGFR3-driven luminal tumors.

### FGFR3 regulates p63 activity through TP63 transcriptomic regulation.

Based on GRN-derived activity scores, we initially predicted that p63 activity decreases upon FGFR3 inhibition using a pan-FGFR inhibitor in FGFR3-dependent BLCa cell lines and increases in FGFR3-S249C–induced mouse bladder tumors (Supplemental Figure 2C and Figure 2A). To independently validate this regulatory link and ensure specificity for FGFR3, we analyzed our previously obtained list of differentially expressed genes (Affymetrix arrays) after FGFR3 knockdown (KD) using siRNAs in RT112, MGH-U3, and UM-UC-14 cells (9, 27) and comparing FGFR3-S249C–induced mouse bladder tumors’ transcriptomic data with those from normal urothelium (23).

**Figure 4:**
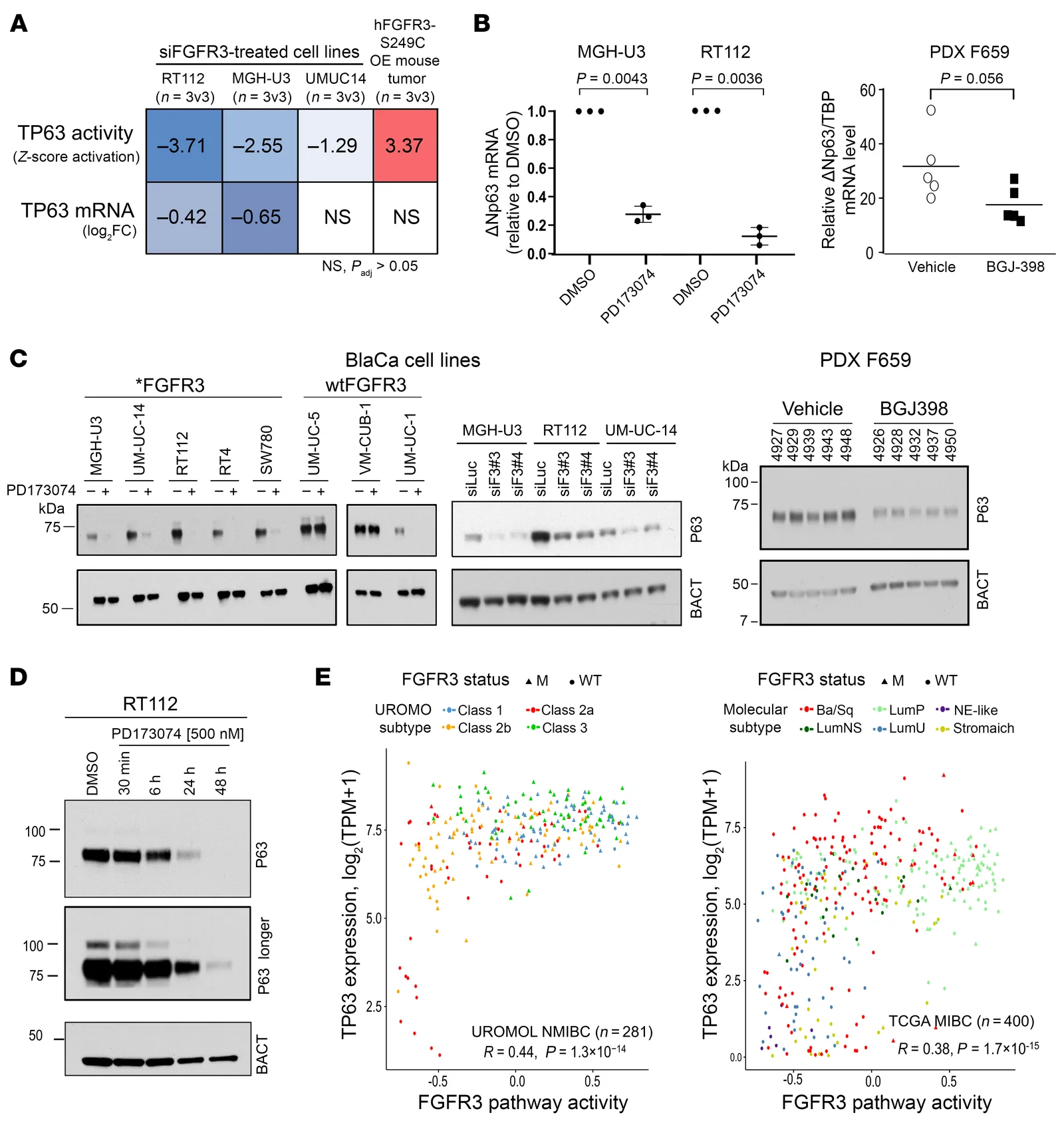
FGFR3 regulates p63 activity through *ΔNp63* transcriptional regulation. (**A**) Upstream regulator analysis using Ingenuity Pathway Analysis software (IPA) identifying p63 as inhibited following *FGFR3* knockdown in FGFR3-dependent cell lines and activated in *FGFR3*-S249C–induced mouse bladder tumors. siFGFR3, FGFR3 siRNA; hFGFR3-S249C OE, human *FGFR3*-S249C overexpression. (**B**) ΔNp63 mRNA expression following FGFR3 inhibition in FGFR3-dependent bladder cancer cell lines (PD173074) for 48 hours (*n* = 3 independent experiments) (left) and in an *FGFR3*-S249C PDX (BGJ398, 30 mg/kg/d, 4 days) (*n* = 5 mice/group) (right). Statistical comparisons were performed using an unpaired 2-tailed *t* test. (**C**) Western blot analysis of p63 levels following siRNA-mediated *FGFR3* knockdown (48 hours) or pharmacological inhibition in *FGFR3*-mutated cell lines (PD173074, 100 nM, 40 hours) (left) and PDX tumors (BGJ398, 30 mg/kg/d, 4 days) (right). (**D**) Time-course analysis of p63 protein expression following FGFR inhibition in RT112 cells (PD173074, 500 nM). (**E**) Spearman’s correlation between *TP63* mRNA expression and FGFR3 pathway activity scores across UROMOL NMIBC and TCGA MIBC cohort, respectively. *FGFR3* mutation status and molecular subtype for each individual tumor are indicated.

Upstream regulator analysis using Ingenuity Pathway Analysis (IPA; QIAGEN) identified TP63 as an inhibited upstream regulator following FGFR3 KD and as an activated regulator in FGFR3-induced mouse tumors (Figure 4A). We observed that FGFR3 inhibition was associated with a significant reduction in TP63 mRNA expression in MGH-U3 and RT112 cells, indicating transcriptional regulation of *TP63* by FGFR3 (Figure 4A). A similar decrease in TP63 mRNA expression associated with a decrease of FGFR3 pathway activity was observed in both TP63-high and TP63-low populations in single-cell RNA-seq data from MGH-U3 cells treated with the FGFR inhibitor erdafitinib (Supplemental Figure 4A). Because *TP63* encodes functionally distinct TAp63 and ΔNp63 isoforms, we next investigated which isoform was regulated downstream of FGFR3. Although our Affymetrix and single-cell RNA-seq data did not allow direct discrimination between isoforms, analysis of our previously published H3K4me3 (trimethylation of histone H3 at lysine 4) and H3K27ac (acetylation of histone H3 at lysine 27), marks of active promoters and enhancers, respectively, ChIP-seq data from BLCa cell lines and tumors (17) revealed active chromatin marks exclusively at the ΔNp63 promoter and enhancer regions (Supplemental Figure 4B). Consistently, RNA-seq–based isoform analysis showed that ΔNp63 was the predominant isoform expressed in BLCa cell lines and human tumors from TCGA cohort, with almost no detectable TAp63 expression (Supplemental Figure 4C). Taken together, these data suggested that FGFR3-driven regulation of p63 activity is ΔNp63 dependent. Accordingly, ΔNp63 mRNA levels decreased after FGFR3 inhibition using a pan-FGFR inhibitor in FGFR3-dependent cell lines (Figure 4B) and in an FGFR3-S249C PDX (F659) (9–11), supporting the relevance of the results in human tumors (Figure 4B). Western blot analysis confirmed reduced p63 protein levels after *FGFR3* KD or FGFR3 pharmacological inhibition in a large panel of *FGFR3*-mutated cell lines (Figure 4C) and in the PDX model (Figure 4C). Long exposures revealed that ΔNp63 was the predominant isoform expressed, though FGFR3 expression or activation also affected very low TAp63 protein levels (Supplemental Figure 4D and Figure 4D). Consistent with the lack of association between p63 activity and FGFR3 pathway activation in basal tumors (Figure 3A), and with the absence of codependency between *TP63* and *FGFR3* in *FGFR3* wild-type luminal cells (Figure 2D), p63 expression was not affected by FGFR inhibition in VM-CUB-1 and UM-UC-5 cells, which represent FGFR3 wild-type, FGFR3-independent basal and luminal models, respectively (Figure 4C). In contrast, p63 expression decreased following FGFR inhibition in UM-UC-1 cells, a luminal FGFR3 wild-type line that shows partial FGFR3 dependency but is also dependent on FGFR2 signaling (Figure 2D and Supplemental Figure 5B; DepMap analysis). Time-course experiments in RT112 cells showed that p63 downregulation occurred only at later time points following FGFR inhibition (≥24 hours) (Figure 4D), supporting transcriptional regulation as the primary mechanism rather than protein stabilization or degradation.

Finally, consistent with these results, analysis of human bladder tumor datasets showed significantly higher TP63 mRNA expression in *FGFR3*-mutated NMIBCs and MIBCs compared with FGFR3 wild-type tumors in UROMOL and TCGA cohorts, respectively (Supplemental Figure 4E). Moreover, as observed for p63 activity (Figure 3, A and B), TP63 mRNA expression was elevated in FGFR3-active NMIBCs and MIBCs independently of the molecular subtypes (Figure 4E).

Although we did not aim here to comprehensively decipher the upstream signaling mechanisms controlling TP63 transcription by FGFR3 but rather focus on studying its unexpected role in a luminal context, we examined whether pathways that we previously demonstrated as implicated in FGFR3-dependent MYC regulation (9) might also contribute to p63 regulation. Western blot analysis of protein lysates from FGFR3-dependent cells treated with the p38 inhibitor (SB203580) or the PI3K inhibitor (LY294002) revealed reduced p63 expression, suggesting that these FGFR3-activated pathways also contribute to the regulation of TP63 expression, whereas the ERK inhibitor (PD98059) had no effect (Supplemental Figure 4F).

### p63 regulates the growth and migration of FGFR3-dependent BLCa cells.

**Figure 5:**
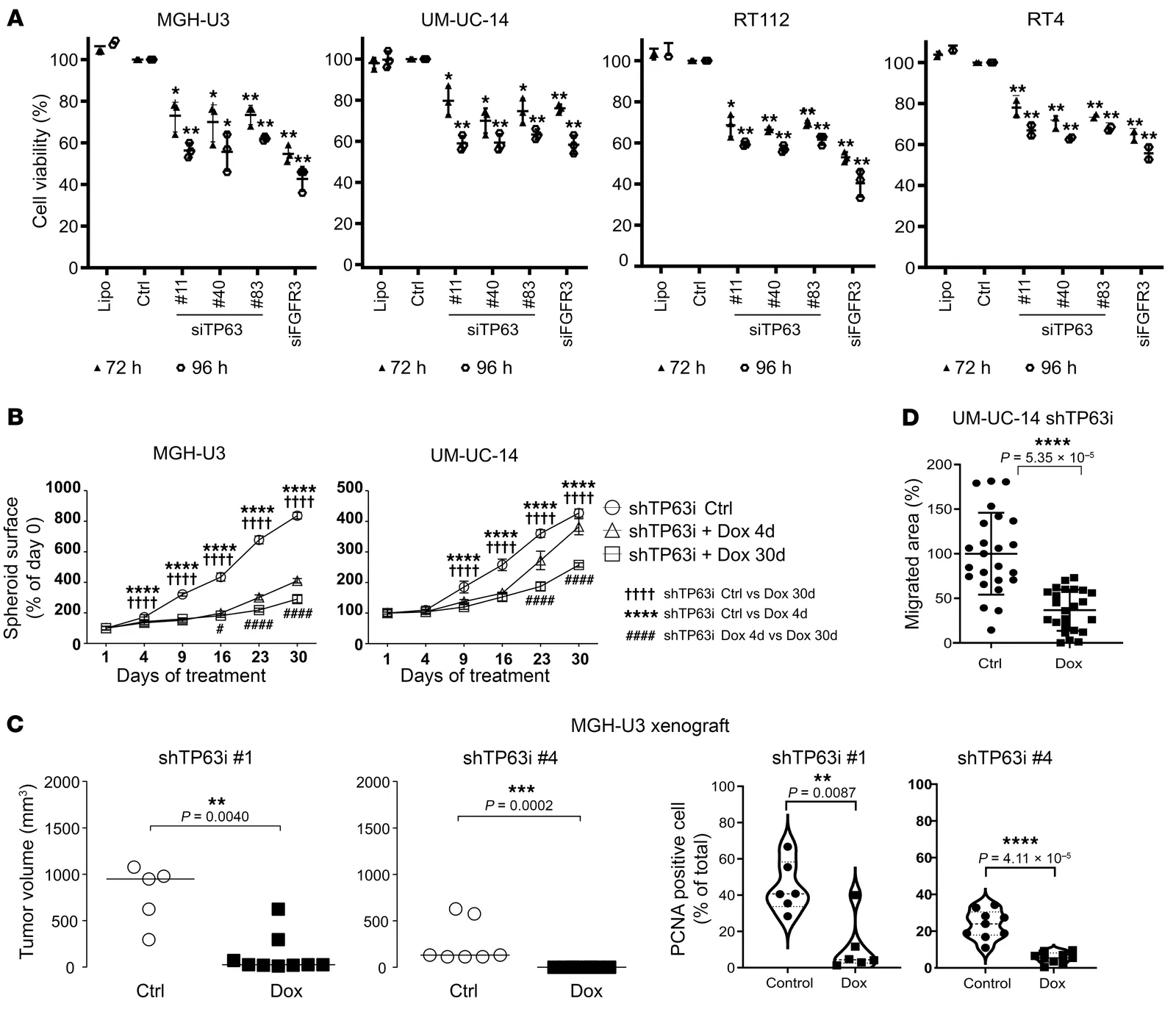
p63 promotes growth and migration of FGFR3-dependent bladder cancer cells. (**A**) Cell viability assessed by CellTiter-Glo following *TP63* siRNA knockdown in FGFR3-dependent bladder cancer cell lines expressing *FGFR3*-mutated proteins (MGH-U3, UM-UC-4) or FGFR3-fusion protein (RT112 and RT4). Results are from 3 independent experiments, and statistical comparisons were performed using an unpaired 2-tailed *t* test. *P ≤ 0.05; **P ≤ 0.01. (**B**) 3D cellular spheroids were established from MGH-U3 and UM-UC-14 cells stably transduced with Dox-inducible shTP63 (shTP63i). Spheroids were grown after a transient (4 days) or with stable (30 days) Dox-induced *TP63* knockdown. Statistical comparisons were performed using 2-way ANOVA. Representative images are shown in Supplemental Figure 5D. ^††††^*P* ≤ 0.0001 (shTP63i Ctrl vs. Dox 30d); ****P ≤ 0.0001 (shTP63i Ctrl vs. Dox 4d); ^#^*P* ≤ 0.05 (shTP63i Dox 4d vs. Dox 30d); ^####^*P* ≤ 0.0001 (shTP63i Dox 4d vs. Dox 30d). (**C**) Xenograft tumors. MGH-U3 cells expressing a Dox-inducible shRNA targeting *TP63* (shTP63i#1, shTP63i#4) were implanted in mice, with or without Dox in drinking water (1 g/L) for 30 days (*n* = 6 to 8 mice per group). Left: Tumor growth was measured twice a week and final tumor volumes were compared. Right: Proliferation was assessed via PCNA immunostaining in tumors. Representative images are shown in Supplemental Figure 5E. Comparisons were performed using Wilcoxon’s test. (**D**) Wound healing assay: migration of UM-UC-14 shTP63i#4 cells after Dox-induced *TP63* knockdown. Migration areas relative to untreated controls are represented. Representative images are shown in Supplemental Figure 5F. Data are expressed as mean ± SDs. Statistical differences were defined by an unpaired 2-tailed *t* test.

To investigate the functional role of p63 in FGFR3-driven luminal tumors, we first sought to validate the dependency of *FGFR3*-mutated BLCa cells on p63 using siRNA-mediated knockdown, as suggested by our analysis of public CRISPR/Cas9 screening data (Figure 2, C and D). We extended these analyses to 2 additional FGFR3-dependent cell lines, RT4 and MGH-U3, expressing FGFR3-S249C and FGFR3-TACC3, respectively. *TP63* was silenced after treatment by 3 siRNAs (Supplemental Figure 5A), resulting in a significant decrease in cell viability across all FGFR3-dependent cell lines tested, 72 hours or 96 hours after transfection (Figure 5A). Consistent with CRISPR-KO–based results (Figure 2D), *TP63* knockdown also reduced viability in basal VM-CUB-1 and L1207 cells but had no effect in luminal FGFR3 wild-type, FGFR3-independent cell lines UM-UC-7 and UM-UC-9, except in UM-UC-1 cells (Supplemental Figure 5B).

To further evaluate the role of p63 in FGFR3-dependent cells in 3D culture and in vivo, we generated stable clones of MGH-U3 and UM-UC-14 cells expressing a doxycycline-inducible (Dox-inducible) shRNA targeting *TP63* (shTP63i). Western blot analysis revealed that Dox treatment induced an approximately 50% reduction in p63 protein levels in both cell lines (Supplemental Figure 5C). Western blot analysis confirmed efficient *TP63* KD upon Dox treatment (Supplemental Figure 5C). In 3D spheroid assays, both continuous and transient (4-day) *TP63* silencing reduced spheroid growth (Figure 5B and Supplemental Figure 5D). Moreover, in vivo, Dox-treated xenografts derived from shTP63i–MGH-U3 cells displayed significantly reduced tumor growth compared with untreated controls (Figure 5C), accompanied by a marked reduction in proliferating cells as assessed by proliferating cell nuclear antigen (PCNA) staining (Figure 5C and Supplemental Figure 5E).

Since *ΔNp63*, the main *TP63* isoform expressed in bladder tumors (Supplemental Figure 4, B and C), is known to regulate cell migration and invasion in several basal/squamous cancer models (28–31), we investigated whether p63 also contributes to FGFR3-induced migration. In a wound healing assay, Dox-induced *TP63* KD in shTP63i UM-UC-14 cells significantly impaired cell migration, with effects comparable to those observed after FGFR3 inhibition via a pan-FGFR inhibitor (Figure 5D and Supplemental Figure 5F).

### p63 directly regulates genes involved in proliferation and luminal differentiation and establishes an FGFR3-p63 feedback loop.

**Figure 6:**
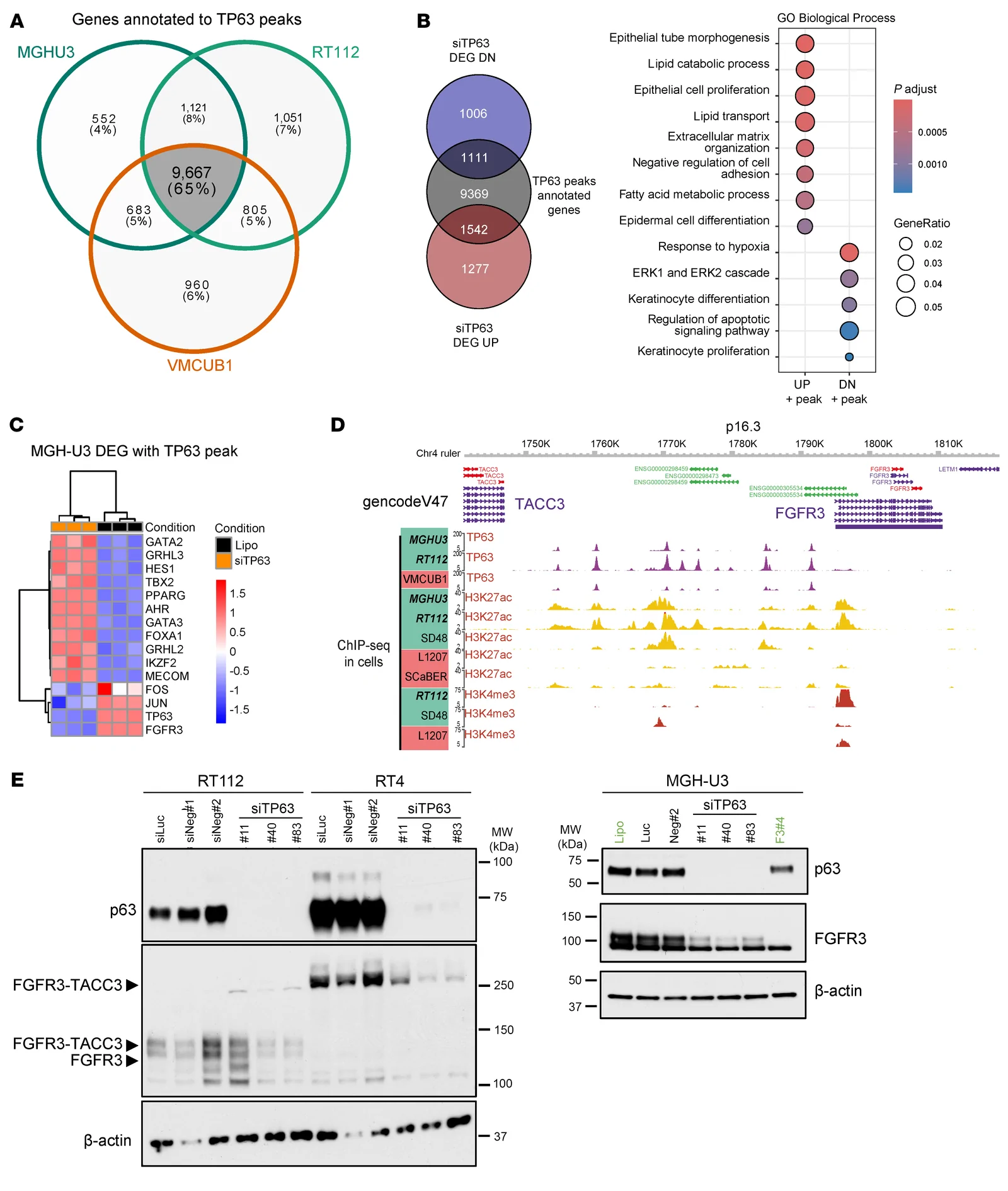
p63 directly regulates proliferation and differentiation genes and establishes an FGFR3–p63 feedback loop. (**A**) Overlap of p63-bound genes identified by ChIP-seq in luminal FGFR3-dependent cell lines (MGH-U3 and RT112) and in basal cell lines (VM-CUB-1). (**B**) Venn diagram comparing genes associated with a p63 peak and genes differentially regulated upon siRNA-mediated knockdown of *TP63* in MGH-U3. The intersections are considered p63 direct targets (left). Gene Ontology enrichment analysis of directly repressed and activated p63 target genes in FGFR3-dependent luminal MGH-U3 cells (right). (**C**) Heatmap visualization of expression changes after *TP63* KD for selected p63-regulated direct target genes: focus on luminal differentiation TFs and FGFR3. Lipo, Lipofectamine (transfection control). (**D**) TP63 and chromatin mark (H3K27ac, H3K4me3) occupancy at *FGFR3* regulatory regions in bladder cancer cell lines. Basal cell lines are highlighted in red, luminal cell lines in green. (**E**) Western blot validation of FGFR3 downregulation following siRNA-mediated *TP63* knockdown (48 hours) in RT112 and RT4 cells expressing FGFR3-TACC3 and in MGH-U3 cells (FGFR3-Y375C). β-Actin was used as a loading control.

To uncover the transcriptional program controlled by p63 in *FGFR3*-mutated luminal cells, and compare it with classical basal contexts, we integrated p63 ChIP-seq data with RNA-seq data after *TP63* KD in MGH-U3, RT112, and VM-CUB-1 cells. Annotation of ChIP-seq peaks to the nearest genes identified 14,839 unique p63-bound genes, with 9,667 (65%) shared across all 3 cell lines (Figure 6A and Supplemental Figure 6, A and B). This highly significant 3-way overlap (hypergeometric test, *P* ≈ 5 × 10^–131^) indicated a conserved set of p63-bound regulatory regions across luminal and basal contexts. We next defined p63-dependent targets in FGFR3-driven tumors by integrating ChIP-seq binding with genes differentially expressed after *TP63* KD. Among the 4,936 TP63-regulated genes (DESeq2 analysis, |log_2_FC| > 0.58) (Supplemental Table 3), 2,642 were direct p63 targets, including 1,542 upregulated and 1,111 downregulated genes (Figure 6B). Gene Ontology (GO) enrichment analysis in MGH-U3 revealed that direct p63 targets were enriched for pathways involved in cell differentiation, proliferation, and lipid metabolism (Figure 6B), consistent with processes regulated by p63 in VM-CUB-1 basal cells (Supplemental Figure 6C) and by FGFR3 (23).

In line with p63 association with basal features in FGFR3-driven tumors even in a luminal context (Figure 3), several key TFs for luminal differentiation, including FOXA1, GATA3, PPARG, and AHR (16, 17, 19), were upregulated following *TP63* KD and identified as direct p63 targets (Figure 6C and Supplemental Table 3). Notably, *FGFR3* itself was a direct and positively regulated p63 target (Figure 6C), revealing a positive feedback loop between FGFR3 signaling and p63. ChIP-seq for p63 and active chromatin marks in RT112 and MGH-U3 confirmed p63 binding at *FGFR3* regulatory regions through p63 binding at enhancer regions (Figure 6D). Western blot analysis in RT112, MGH-U3, and RT4 validated decreased FGFR3 protein levels following *TP63* KD (Figure 6E).

Together, these results identify p63 as a key transcriptional regulator in FGFR3-dependent luminal tumors, controlling genes involved in proliferation and differentiation and participating in a positive feedback loop reinforcing FGFR3 signaling.

## Discussion

We present a BLCa-GRN, inferred de novo through a reverse engineering method, without relying on prior knowledge. Identifying this network of TFs/coTFs is particularly important because disease phenotypes, including those related to disease progression and response to therapy, have been demonstrated to be maintained by small groups of TFs and coTFs (32, 33). Many of the regulators forming part of this network, such as FOXA1, PPARG, GATA3, and p63, were previously associated with BLCa and urothelial differentiation (3, 6, 34), reinforcing the biological significance of the inferred BLCa-GRN. However, we also uncovered potentially novel TFs, including key TFs regulating the viability of *FGFR3*-mutated tumor cells, such as FOXM1. Nonetheless, our BLCa-GRN did not identify some previously described key regulators of *FGFR3*-mutated BLCa, such as ESR1 (23) and MYC (9). This failure arises because the algorithm used infers a GRN only from TFs/coTFs that have a significant variation in expression across the samples in the input data. This unavoidable limitation, inherent to other GRN reconstruction algorithms (35), stresses the need to integrate multiple bioinformatics and experimental approaches for a more comprehensive GRN analysis. While this study employed only one bioinformatics strategy, we leveraged transcriptomic data originating from both patient samples and experimental models, strengthening our findings.

Our analysis revealed that FGFR3 activated different TFs/coTFs depending on the molecular tumor subtype, which may have some therapeutic implications. Among the TFs regulated by FGFR3 across all datasets, including LumP (where mutations of *FGFR3* are enriched), we identified p63. While the role of p63 has been well characterized in BLCa in a basal molecular context (21, 24–26), its involvement in *FGFR3*-mutated tumors remained poorly explored. Notably, while this hyperactivation of the p63 regulon has already been described in LumP MIBC (3), and high *TP63* expression has been associated with *FGFR3* mutation and UTX (ubiquitously transcribed tetratricopeptide repeat X-linked; also known as KDM6A) (36), no direct link between FGFR3 activity and p63 expression has been established.

In this study, we demonstrated that FGFR3 regulates p63 expression in both BLCa cell lines and PDX models. We further showed that p63 mediates the proliferation and migration of FGFR3-dependent BLCa cells both in vitro and in vivo. Additionally, we identified a positive feedback loop between FGFR3 and p63, similar to what we previously described for FGFR3 and MYC (9). These results suggest a potential therapeutic strategy, as targeting p63 could disrupt FGFR3 expression and help prevent resistance to FGFR inhibitors. The emerging development of degraders to inhibit master regulators allows us to consider them as promising therapeutic targets. We did not decipher extensively the signaling pathways leading to p63 activation by FGFR3 but only showed the contribution of p38 and AKT as already shown for MYC regulation by FGFR3. A better characterization would be worth it to identify alternative targets within this upstream regulatory pathway, including chromatin modifiers or TFs binding regulatory elements of *ΔNp63*.

Interestingly, p63 is known to drive an invasive program in the more aggressive basal BLCa subtypes (21, 26, 37, 38), yet it appears to regulate cell migration in an *FGFR3-*altered context, typically associated with NMIBCs or luminal-like MIBCs. However, this observation could align with the fact that luminal *FGFR3*-mutated tumors are less differentiated than other luminal tumors (36, 39), with FGFR3 activation cooperating with UTX loss to promote basal differentiation programs, in luminal bladder tumors. Consistently, our results showed that FGFR3 inhibition reduced the basal differentiation in *FGFR3*-mutated cells. Using single-cell data from our PDX and BLCa cell line models, we further demonstrated that p63 activation by FGFR3 was heterogeneous, independent of proliferation rate but associated with the basal differentiation. Our preliminary investigations to evaluate the spatial activation of p63 suggested that this heterogeneous activation was not associated with basal cell layering (data not shown), but this hypothesis warrants further investigation to be definitively excluded. However, this supports the notion that p63 is associated with basal differentiation even in FGFR3-dependent tumors presenting an overall luminal phenotype.

Our work provides a BLCa-specific GRN, and we focused here on the identification within this GRN of TFs and coTFs regulated by FGFR3 and essential in *FGFR3*-mutated tumors. We have further validated the function of p63, one of the putative essential regulators activated by FGFR3 in bladder tumors. However, such GRNs could be further studied to improve current therapeutic options and increase our understanding of BLCa biology. For example, the role of the second key element identified in the FGFR3-GRN network, FOXM1, would be worth being elucidated, as high expression of this TF is related to a poor prognosis in BLCa (40, 41).

## Methods

### Sex as a biological variable

Xenograft studies were performed in male nude mice using MGH-U3 cells and the F659 PDX, as both models were established from male patients with *FGFR3*-mutated BLCa. For the UPII-hFGFR3-S249C transgenic mouse model, tumors were obtained from both male and female mice. Cell lines used for in vitro experiments were derived from both male and female patients with BLCa. For human tumors, both male and female patients were included in the UROMOL and TCGA cohorts, and no sex-specific differences in FGFR3-ΔNp63 regulation were observed.

### Inference of the GRN

As a first step, a BLCa-specific GRN was constructed from the CCLE human BLCa cell line transcriptome (*n* = 36 BLCa cells) (253, 5637, 639V, 647V, BC3C, BFTC905, CAL29, DSH1, HT1197, HT1376, J82, JMSU1, KBMC2, KU1919, RT112, RT11284, RT4, Scabber, SLR20, SW1710, SW780, T24, TCCsup, UM-UC-1, UM-UC-10, UM-UC-11, UM-UC-13, UM-UC-14, UM-UC-16, UM-UC-3, UM-UC-4, UM-UC-5, UM-UC-6, UM-UC-9, VM-CUB-1, UBLC1) using the Bioconductor CoRegNet package (14). The CoRegNet package implements the H-LICORN algorithm (13) to infer a series of GRNs from transcriptomic data and a list of previously defined regulators. The list of known regulators (TFs/coTFs; *n* = 2,375) is defined from previously published datasets (42, 43). In summary, H-LICORN infers the best GRN that describes the regulatory interactions between regulators and their target genes through 4 steps: (a) First, the transcriptomic matrix is discretized into –1, 0, and 1 values that fit its per-gene distribution of expression. In addition, genes present in the transcriptome matrix are classified into regulators and target genes, and only those presenting significant variation in expression levels across samples are retained. (b) Second, potential sets of coactivators and corepressors regulating the expression of a target gene are determined through frequent item search techniques. (c) Third, for each target gene, a list of candidate coactivators and coinhibitor sets (GRNs) is selected by employing an association rule metric (based on gene regulation). (d) Next, such sets of GRNs are scored following a regression model between the expression of the regulators forming part of the GRN set and the expression of their target genes. The top 10 GRN candidate sets presenting the best R2 scores for each target gene are retained. During this GRN reconstruction, we applied the Benjamini-Hochberg correction and retained only interactions that passed a stringent FDR threshold of 1% (FDR < 0.01).

CoRegNet refines the inferred GRN by incorporating extensive published interaction evidence from various bioinformatics tools and databases. This includes 1,107,092 TFBS interactions (ChEA2, ENCODE ChIP v3, Motif Db, HOCOMOCO, ITFP, TRRUST, PWMEnrich, and targets) and 1,149,582 PPIs (iRefR, BioGrid, HIPPIE, STRING, and HPRD). For this step, we assessed the enrichment of inferred regulatory interactions against 2 independent gold standard datasets — PPIs and TFBS — using Fisher’s exact tests. Because this validation involved only 2 global enrichment tests, the multiple-testing burden was minimal. Importantly, FDR-adjusted *P* values remained extremely significant (*P* < 1 × 10^–100^).

Each GRN is given a score that merges the previous *R*^2^ score and a score representing validated regulatory interactions. The GRN with the maximum final merged score is selected and then transformed into a coregulatory network based on the target genes shared between the inferred regulators.

### Estimation of sample-specific TF/coTF activity

Using the CoRegNet package, we computed a network-based regulatory influence representing an estimated activity for each TF/coTF with enough gene targets for each transcriptome sample. Briefly, the measure of influence estimates the activity of a TF/coTF based on a Welch *t* test comparing the distribution of expression of the set of activated and repressed target genes for each TF/coTF in each sample. In addition, an advantage of the CoRegNet package is that it is possible to compute the TF/coTF influence for many datasets via the regulatory information of the same GRN. In this study, we constructed a BLCa-GRN and then calculated the influence of the inferred TF/coTFs via transcriptomic data from different sources.

### Identification of FGFR3-driven GRNs

Using the inferred BLCa-specific GRN, we calculated the influence of the predicted TFs/coTFs using transcriptomic data from preclinical models in which the activity or gene expression of FGFR3 was altered. The first dataset used was the E-MTAB-4749 transcriptomic data from MGH-U3 (FGFR3-Y375C) and RT112 (FGFR3-TACC3) BLCa cell lines treated with the FGFR pan-inhibitor AZD4547 (100 nM, 2, 6, 24 hours) (22). The second dataset was the human ortholog transcriptomic data of FGFR3-induced murine bladder tumors (murine model of hFGFR3-S249C overexpression in the urothelium) (23). The most influential TFs/coTFs that presented opposite and coherent activity between the FGFR3-inhibited and FGFR3-overexpressing preclinical models were taken as FGFR3-driven regulators.

### Visualization of the GRNs

Visualization of the constructed networks and overlay of the computed influence and regulatory interactions were performed using Cytoscape (44).

### Cell culture

Human BLCa-derived cell lines were obtained from different repositories: RT112, UM-UC-14, UM-UC-7, UM-UC-9, SW-780, and VM-CUB-1 were obtained from DSMZ; UM-UC-5 cells were obtained from the European Collection of Authenticated Cell Cultures collection; and Francisco X. Real, CNIO, Madrid, Spain, supplied MGH-U3 and RT4 cells. MGH-U3 and UM-UC-14 harbor Y375C and S249C FGFR3 mutations, respectively. RT112, SW-780, and RT4 expressed the FGFR3-TACC3 fusion protein. UM-UC-5 and VM-CUB-1 express wild-type FGFR3. MGH-U3, UM-UC-14, SW-780, UM-UC-5, UM-UC-9, and UM-UC-7 cells were cultured in DMEM, whereas RT112 and RT4 cells were cultured in RPMI. All culture media were supplemented with 10% FBS. Cell culture was carried out at 37°C under a 5% CO_2_ atmosphere.

### FGFR3 inhibition

MGH-U3, RT112, RT4, SW-780, UM-UC-14, UM-UC-5, UM-UC-1, and VM-CUB-1 cells were seeded in 100 mm plates at the following total densities: 5.0 × 10^6^, 4.0 × 10^6^, 4.5 × 10^6^, 1.8 × 10^6^, 3.0 × 10^6^, 5.0 × 10^6^, 1.8 × 10^6^, and 0.8 × 10^6^ cells/100 mm dish, respectively. The cells were plated and allowed to adhere overnight. A panel of bladder cancer cells were treated for 40 hours with the pan-FGFR inhibitor PD173074 (100 nM) (Calbiochem, Merck Eurolab). RT112 cells were treated for 30 minutes, 6 hours, 24 hours, or 48 hours with PD173074 (500 nM). The control cells were treated with a DMSO vehicle and diluted the same way as the inhibitor. At the end of treatment, whole-cell lysates or nuclear and cytosolic cell fractions were recovered for immunoblotting. Cellular fractions were obtained using a Thermo Fisher Scientific NE-PER nuclear and cytoplasmic extraction kit (catalog 78833) according to the manufacturer’s protocol.

Protein lysates (20 μg) previously extracted from a PDX (F659) bearing the FGFR3-S249C mutation in mice treated with or without the pan-FGFR inhibitor BGJ398 (30 mg/kg/d; 4 days) were used for immunoblotting (9).

### Gene knockdown and cell viability assays

MGH-U3, RT4, RT112, UM-UC-14, VM-CUB-1, UM-UC-5, UM-UC-9, and UM-UC-7 cells were transfected for 48, 72, or 96 hours with 5 nM siRNA and Lipofectamine RNAi Max reagent (Invitrogen) as indicated in the manufacturer’s protocol. For protein or RNA analysis, cells were plated in 6-well plates at a seeding density of 300,000 cells/well, and the cells were lysed at 48 hours after transfection with the appropriate lysis buffer. For the cell viability assays, the MGH-U3 cells were plated in 96-well plates at a seeding density of 10,000 cells/well, the other cells were plated at a density of 5,000 cells/well, and the cell viability was measured (CellTiter-Glo, Promega) at 72 and 96 hours.

Three *TP63* siRNAs (*TP63* siRNAs #11, #40, and #83; Ambion Silencer Select, Thermo Fisher Scientific) were used, and a siRNA targeting *FGFR3* was used as a positive control for FGFR3-dependent cell lines (QIAGEN). As negative controls, we used a siRNA directed against luciferase (QIAGEN SI03650353) and the nontargeting negative control Silencer Select (Thermo Fisher Scientific 4390846). The sequences of the siRNAs used are provided in Supplemental Table 4.

### Real-time reverse transcription quantitative PCR

RNA from BLCa cell lines was extracted with QIAGEN’s RNeasy Mini Kit according to the manufacturer’s protocol. RNA from our human bladder tumor cohort was extracted through cesium chloride density centrifugation, as described previously.

Reverse transcription was performed with 1 μg of total RNA using a high-capacity cDNA reverse transcription kit (Applied Biosystems). cDNAs were subsequently amplified via PCR in a Roche real-time thermal cycler with the Roche TaqMan Master mix and Master probe primers listed Supplemental Table 4.

### Immunoblotting

Protein extraction from the MGH-U3, RT112, RT4, SW-780, UM-UC-14, UM-UC-5, UM-UC-1, and VM-CUB-1 cell lines was performed through cell lysis in Laemmli buffer (50 mM pH 6.8 Tris-HCl, 2.5 mM EDTA, 2.5 mM EGTA, 2 mM DTT, 5% glycerol, 2% SDS) supplemented with protease inhibitors and phosphatase inhibitors (Roche). Following clarification of the cell lysates by centrifugation at 10,000*g*, protein levels were quantified with a BCA protein assay (Thermo Fisher Scientific). Ten micrograms of whole-cell lysate and 5 μg of cell fractionation lysate were resolved by SDS-PAGE in 7.5% or 15% polyacrylamide gels, depending on the molecular weight of the proteins to be analyzed. The gels were electrotransferred onto nitrocellulose membranes (Bio-Rad), and protein transfer was verified by amido black staining before immunoblotting. Membranes were cut according to molecular weight before antibody incubation when appropriate. Proteins were detected with antibodies against p63 (Abcam ab5309, 1:4,000 dilution), MYC (Cell Signaling Technology 9402, diluted 1:1,000), and FGFR3 (Abcam ab133644, diluted 1:5,000). α-Tubulin and β-actin (Sigma-Aldrich references T6199 and A2228, respectively; both diluted at 1:20,000) were used as loading controls. The secondary antibodies used were HRP-linked anti-mouse IgG and anti-rabbit IgG (Cell Signaling Technology references 7076 and 7074, respectively, diluted 1:3,000).

shTP63i cells were plated in 60 mm plates and treated with or without Dox for 72 hours. Protein was then extracted using RIPA-EDTA and protease cocktail inhibitor. The protein concentration was measured via the Bradford method (Merck Millipore 1103060500). Proteins (50–80 μg) were resolved on polyacrylamide gels; transferred to PVDF membranes; incubated with antibodies against p63 (Abcam ab53039, diluted 1:1,000), β-actin (Sigma A5441, diluted 1:10,000), and MT1-MMP (Santa Cruz Biotechnology sc-30074, diluted 1:1,000); and visualized via a LI-COR C-DiGit Blot Scanner. Images were analyzed using Gel Pro Analyzer software (Media Cybernetics).

### RNA-seq

For the whole-genome profiling experiment, MGH-U3 and VM-CUB-1 cells were transfected for 48 hours with *TP63* siRNA #11 (as described above).

Triplicate RNA isolates from siTP63-transfected and control cells (Lipofectamine RNA iMax, Invitrogen) were prepared using the QIAGEN RNeasy Mini Kit supplemented with DNase treatment, and the RNA sample quality was controlled with the Agilent 2100 Bioanalyzer system. RNA-seq was carried out on stranded mRNA (1 μg) with an Illumina NovaSeq S1 sequencing system at a sequencing depth of 30 million reads per sample for MGH-U3 and using Illumina NovaSeq 6000 S1 SR100 after QuantSeq 3’mRNA library prep kit FWD extraction for VM-CUB-1. Quality control and data filtering were done using FastQC (Babraham Bioinformatics Institute). Filtered reads were mapped to the hg19 human genome and annotated via the STAR aligner. Statistically significant differences in gene expression were determined by performing a differential analysis using DESeq2. The Benjamini-Hochberg (FDR) method adjusted the *P* values for multiple testing.

### p63 ChIP-seq

MGH-U3, RT112, and VM-CUB-1 cells were cross-linked in 1% formaldehyde for 10 minutes at room temperature. The reaction was stopped with glycine (final concentration of 125 mM, 5 minutes’ incubation at room temperature). Fixed cells were washed twice with PBS and harvested with a cell scraper. Following centrifugation (10,000*g* for 5 minutes), the cell pellet was resuspended in extraction buffer (250 mM sucrose, 10 mL Tris-HCl pH 8, 10 mM MgCl_2_, 1% Triton X-100, and 5 mM β-mercaptoethanol) supplemented with protease inhibitors (Roche). The cells were centrifuged at 3,000*g* for 10 minutes, and the recovered samples were analyzed using the ChIP-IT High Sensitivity Kit (Active Motif, 53040). ChIP was carried out in duplicate using a p63 antibody (Cell Signaling Technology, D2K8X XP, 13109). Sequencing was carried out in collaboration with the GenomEast platform of the IGBMC Strasbourg, a member of the “France Génomique” consortium (ANR-10-INBS-0009). p63 ChIP and publicly available datasets were processed as follows. Sequencing adapters were first removed using TrimGalore (v0.6.6, --paired, --stringency 6), and then resulting reads were mapped to the hg19 genome using bowtie2 (v2.4.2) (45), reporting at most 1 hit for each read (the best one according to Mapping Quality score). Peaks were called using MACS2 (v2.2.7.1) to capture narrow (-*q* 0.05) or broad peaks (-*q* 0.05 --broad --broad-cutoff 0.1). A ChIP with IgGs was used as a control for each condition, and bedtools (v2.31.1) was used to keep overlapping peaks between replicates. Tracks showing average profile were generated after merging replicates (except for p53 ChIP in RT112, only 1 sample) from BAM files using samtools (v1.21) before applying RPKM normalization using deeptools (3.5.6). The peak regions were annotated to genes using annotatePeak function of ChIPseeker package (1.42.1) (46). To determine direct p63 targets, we intersected genes differentially regulated upon knockdown with genes annotated to a p63 peak in each cell line.

### GO enrichment

To perform the enrichment analysis, the R package clusterProfiler was used to identify biological processes (GO-BPs) enriched in the set of p63 target genes. Significantly enriched GO-BP terms were those with an adjusted *P* value (Benjamini-Hochberg) ≤ 0.05.

### Cell spheroid culture

3D cell cultures were generated by the hanging-drop seeding method; 3 × 10^3^ cells were seeded in 20 μL of complete medium for 72 hours (MGH-U3) or 96 hours (UM-UC-14) and then plated on agar-coated, 96-well plates. The culture medium with or without Dox was completely replaced twice weekly, and images were taken weekly. The diameter was measured via ImageJ software (NIH), and the surface area was subsequently calculated. After 30 days, the spheroids were fixed in methacarn and embedded in paraffin, sliced, and immunostained via a standard immunofluorescence method. p63 was stained with the primary antibody CM163B (Biocare Medical) and the secondary antibody Alexa Fluor 488 (Abcam ab150113). Nuclei were stained with DAPI (Cas28718-90-3, Sigma-Aldrich).

### Wound healing assay

Cells were seeded in 6-well plates and treated with or without Dox for 72 hours. Two wounds were performed in each well, and then a PBS wash was performed to eliminate the released cells. The culture medium was replaced with a 2% FBS medium. Pictures were taken immediately (t0) and 24 hours later (t1). The wound area was measured in both situations via ImageJ, and the migrated area was calculated using the formula (At1*100)/At0 and then normalized to the control.

### Mouse UPII-hFGFR3-S249C transcriptome

We used Affymetrix Mouse Exon 1.0 ST array transcriptomic data of tumor samples from a previously established FGFR3-induced murine model of bladder tumors (23). Data were available for control urothelium, FGFR3-S249C–induced hyperplasia, and bladder tumors.

### TP63 knockdown in vivo (cell line xenografts)

Human BLCa *TP63*-silenced cells were injected subcutaneously into the right flank of 20 male nude mice (2 × 10^6^ cells in 100 μL of PBS). When the tumors were palpable (1 mm × 1 mm), 10 mice received 1 g/L of Dox in the drinking water (Dox group), and 10 mice received only water (Ctrl group). Tumors were measured using a caliper twice a week, and tumor volume was calculated via the following formula: 3/4π × (largest diameter) × (shorter diameter)^2^. At the end of the experiment, the mice were sacrificed, and the tumors were removed, fixed in methacarn, and subsequently paraffin-embedded for sectioning and immunostaining. p63 was labeled with the primary antibody CM163B (Biocare Medical, dilution 1:100) and the secondary antibody Alexa Fluor 546 (A110003, dilution 1:1,000). PCNA was detected with a primary antibody (2586, Cell Signaling Technology, dilution 1:100) and secondary Alexa Fluor 488 (ab150113, Abcam, dilution 1:1,000). Nuclei were stained with DAPI (Cas28718-90-3, Sigma-Aldrich).

### PDX

PDXs from F659 models were obtained as previously described (11), with all procedures already approved at Urosphere. Fresh tumors were used for single-cell RNA-seq analysis.

### Single-cell RNA-seq

MGH-U3 cells treated with DMSO or 100 nM erdafitinib for 38 hours and PDXs from the F659 model bearing FGFR3 mutations (11) were sequenced via single-cell RNA-seq. A total of 10,000 cells were loaded per sample using the single-cell 3’ Reagent Kit protocol (10x Genomics), and the cells were sequenced at a mean depth of 50,000 using a NovaSeq 6000. The sequencing output was processed using Cell Ranger from 10x Genomics (47). The data pretreatment was performed with the Seurat v5 R package (48) using the filtered matrix from the Cell Ranger output. For the F659 PDX, the cell cycle was corrected via SCTransform, and the cells were clustered to distinguish between humans and mice. Mice and low-quality human cells were removed, and cells with 5%–35% human mitochondrial genes, a minimum of 1,000 counts, and more than 10% ribosomal genes were removed. The p63 and FGFR3 signatures from Supplemental Table 1 and our previous study (23) were computed, as were the Ba/Sq signatures via the centroids from the MIBC classification consensus (3).

For the MGH-U3 DMSO and erdafitinib datasets, cells with more than 10% mitochondrial genes and fewer than 15,000 counts were removed. Pretreatment was performed, and the DMSO and erdafitinib datasets were integrated using Reciprocal PCA. The p63, FGFR3, and Ba/Sq signatures were also computed.

### Statistics

All in vitro experiments were independently carried out 2 or 3 times, each performed in triplicate. In vivo experiments used 6 to 8 mice per group. The data are presented as the means ± SDs. Unpaired 2-tailed *t* test, 2-way ANOVA, and Wilcoxon’s test were used for comparison. Correlation analyses between gene expression signatures, including FGFR3 pathway activation and TP63-associated signatures across patient cohorts, were performed using Spearman’s rank correlation coefficient. For microarray data analysis, linear models for microarray data (LIMMA) (49) R package was used, and *P* values were adjusted via the Benjamini-Hochberg method. A corrected *P* value of less than 0.05 was considered statistically significant.

### Study approval

To establish cell line xenografts, nude male mice were obtained from CNEA (Comisión Nacional de Energía Atómica, Argentina). The CICUAL (Comité Institucional para el Uso y Cuidado de Animales de Laboratorio, Argentina) approved all the procedures (Instituto de Oncología A.H. Roffo; Protocol number 2017/03, Buenos Aires, Argentina). Tumors from PDX model F659 were obtained by Urosphere (Toulouse, France), and experimental protocols were reviewed by CEEA-122 Ethical Committee for Protection of Animals used for Scientific Purposes and approved by French Ministry for National Education, Higher Education and Research, Paris, France, under the number *APAFIS#14811-2018042316405732* *v4*. Human sample datasets were obtained from the previously published UROMOL (4) and TCGA (6) cohorts, for which informed consent and ethical approval had been obtained.

### Data availability

#### Public data collection.

The human BLCa cell transcriptome (RNA-seq) and *FGFR3* mutational status corresponding to 36 BLCa cell lines (5 cell lines in which *FGFR3* was mutated and whose growth was dependent on FGFR3 signaling) were collected from the CCLE (DepMap 2019Q1, Broad Cancer Dependency Map Project) (50).

The bladder tumor transcriptome (RNA-seq) was collected from 2 large cohorts of patients with NMIBC and MIBC. The NMIBC transcriptome and *FGFR3* mutational status data were collected from the published dataset by Hedegaard et al. (UROMOL cohort, ArrayExpress E-MTAB-432) (4) and corresponded to 476 tumors (272 of which had mutated *FGFR3*). RNA-seq data for the MIBC cohort were collected from TCGA dataset (cbioPortal) (6) of 408 tumors (52 tumors presented a mutated *FGFR3*).

Gene invalidation (CRISPR/Cas9; CERES dependency score) large screen data to identify essential genes in human cancer cell lines (27 BLCa cell lines) were obtained from the AVANA genetic dependency dataset (AVANA 2019Q3, Achilles Project, Broad Institute) (15).

Transcriptomic data (human Affymetrix DNA Array U133 Plus 2) of MGH-U3 and RT112 cells treated with AZD459 [100 nM] were recovered from the Array Express E-MTAB-4749 dataset (22).

Transcriptomic data (mouse Affymetrix DNA array exon 1st) of UII-FGFR-S249C transgenic mouse bladder tumors and normal urothelium from littermate mice were obtained from NCBI GEO GSE151888 (23).

Histone mark ChIP-seq data from bladder cancer cell lines and tumors were previously published (17) and deposited under the following accession numbers: GSE193889 (tumor ChIP-seq), GSE193886 (bladder cancer cell lines), and GSE195768.

#### New data.

The transcriptomic data of MGH-U3, VM-CUB1, and RT112 cells after *TP63* siRNA treatment; p63 ChIP-seq data for MGH-U3; and single-cell RNA-seq data from B659 PDXs and from MGH-U3 and RT112 cells treated with DMSO or erdafitinib were deposited, respectively, under accessions GSE314629, GSE314908, and GSE315628.

#### Supporting data.

Supporting data values for all figures and supplemental figures are provided in a single Supporting Data Values Excel file, with separate tabs for each figure panel and clear labeling of all data points.

## Author contributions

AMV, MZN, LEV, XM, and JP share first author position, having made significant contributions to this collaborative study involving 3 main laboratories. The order of authorship was determined based on their contributions and leadership in study design, experimental work, and manuscript drafting. AMV conducted experiments, performed bioinformatics analyses, and led the project. MZN carried out experiments, while LEV, XM, and JP performed bioinformatics analyses. LEV and XM revised figures and the manuscript after review. AMV, MZN, LEV, XM, AME, ME, FR, CL, and IBP contributed to study design. AMV, MZN, LEV, XM, MS, FD, KL, HNK, JF, CK, LT, CB, YL, and TY contributed to data acquisition. AMV, MZN, LEV, XM, JP, MS, FD, GG, HNK, CG, MPE, WD, CH, LC, SL, and TY contributed to bioinformatics analysis, data analysis, and interpretation. AME, ME, FR, CL, and IBP share senior authorship, supervising postdocs and PhD students involved in the study within their respective laboratories and contributing to study design. FT, ID, JMP, LD, YA, and PL supervised experiments and analyses conducted in collaboration for this study. IBP coordinated the study and serves as the corresponding author. AMV, MZN, LEV, XM, CL, and IBP drafted figures and prepared the manuscript. All authors contributed to critical review and final manuscript preparation.

## Conflict of interest

The authors have declared that no conflict of interest exists.

## Funding support

- Ligue Nationale Contre le Cancer (Equipe labellisée, recipients: AMV, MS, XM, FD, HNK, JF, LT, CK, CH, LC, YA, FR, and IBP).
- Universidad de Buenos Aires–UBACYT grant 20720190100005BA (recipients: AME and CL).
- Instituto de Salud Carlos III/CIBERONC grant CB16/12/00228 (recipients: JMP, MEP).
- Ministerio de Ciencia e Innovación/FEDER grants PID2019-110758RB-I00 and PID2023-147517OB-I00 (recipient: JMP).
- National Natural Science Foundation of China grants 82303057 (recipient: XM) and 82002672 (recipient: MS).
- Natural Science Foundation of Hubei Province of China grant 2023AFB521 (recipient: XM).
- The Chutian Scholars Program of Hubei Province of China (recipient: XM).
- ITMO Cancer AVIESAN (recipient: XM).
- AMV by a fellowship from the French Ministry of Research.
- MZN by a fellowship from Universidad de Buenos Aires.

## Supplementary Material

Supplemental data

Unedited blot and gel images

Supplemental table 1

Supplemental table 2

Supplemental table 3

Supporting data values

## Version 1 08/03/2026

Electronic publication

## Version 2 10/20/2026

Updated affiliation no. 3
