# Supplementary material for: FGFR3-driven gene regulatory network analysis reveals a protumoral role for p63 in luminal bladder tumors
Source: J Clin Invest. 2026 Aug 3;136(15):e193280. doi: 10.1172/JCI193280 (PMC13430010; doi:10.1172/JCI193280)
Supplement: Supplemental data [file jci-136-193280-s007.pdf]

Supplementary figures

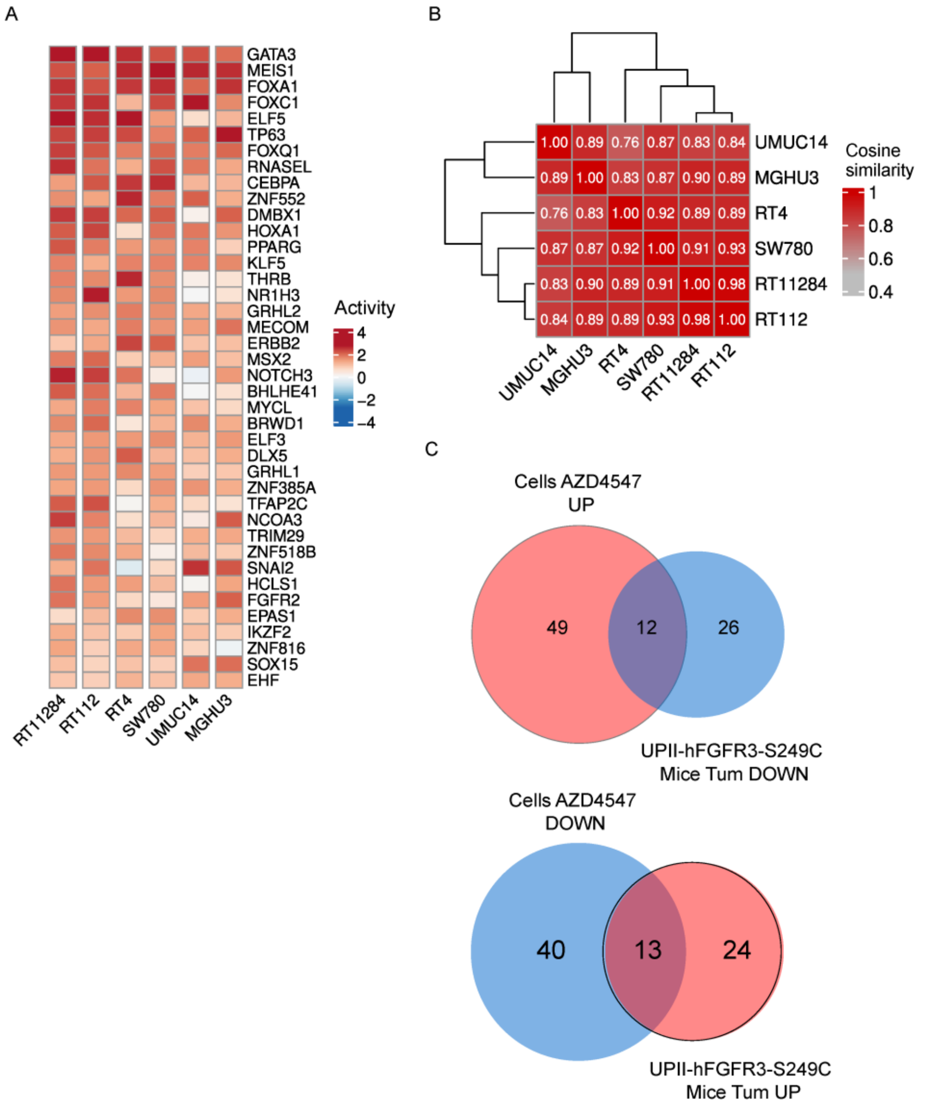

Figure S1: TF activity patterns across *FGFR3*-altered cell lines and cross-species analyses of FGFR3-driven TFs. A) TF/coTF activity scores were computed using the CoRegNet framework in 5 FGFR3-dependent bladder cancer cell lines from the CCLE dataset and MGHU-3 in house transcriptomic data. **B)** Cosine similarity–based clustering of FGFR3-dependent cell lines demonstrating highly concordant TF activity profiles across different *FGFR3* alteration types, (activating point mutations, MGH-U3, UMUC-14 or fusion proteins, RT4, RT112, RT1284, SW780). **C)** Venn diagram of TFs/coTFs of BLCa-GRN with altered activity after FGFR3 perturbations in two transcriptomic datasets: (i) RT112 and MGHU3 treated with a pan-FGFR inhibitor (AZD4547) and (ii) bladder tumors from transgenic mice overexpressing human FGFR3 (S249C) in the urothelium (UPII-hFGFR3-S249C). 25 regulators displayed opposite activation patterns upon FGFR3 inhibition and activation were selected for downstream analyses.

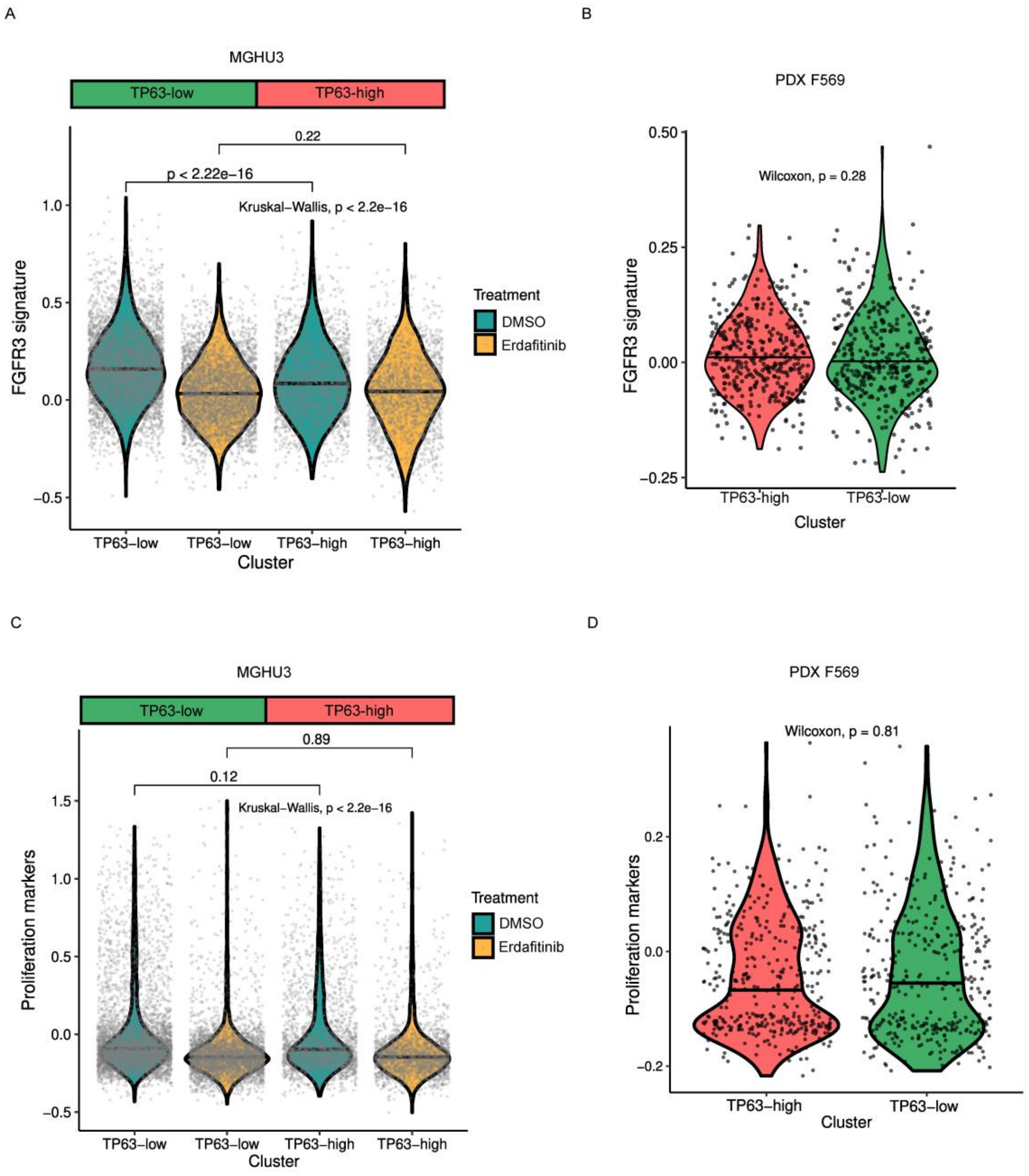

Figure S2: **Single-cell analyses of TP63 heterogeneity in a *FGFR3*-mutated PDX and in MGH-U3 before and after FGFR3-inhibition.**

A-B) FGFR3 activation signature levels across clusters identified in MGH-U3 (Figure 3D) and in *FGFR3*-S249C PDX model (F659) (Figure 3C) (B), showing a decrease after erdafitinib treatment (C). **C-D)** Proliferation markers across clusters in MGHU-3 (C) and in PDX F659 (D) and showing a decrease after erdafitinib treatment. **A-D)** Comparisons between clusters were performed using the Kruskal–Wallis test. Comparisons between TP63-low and TP63-high clusters were performed using the Wilcoxon rank-sum test.

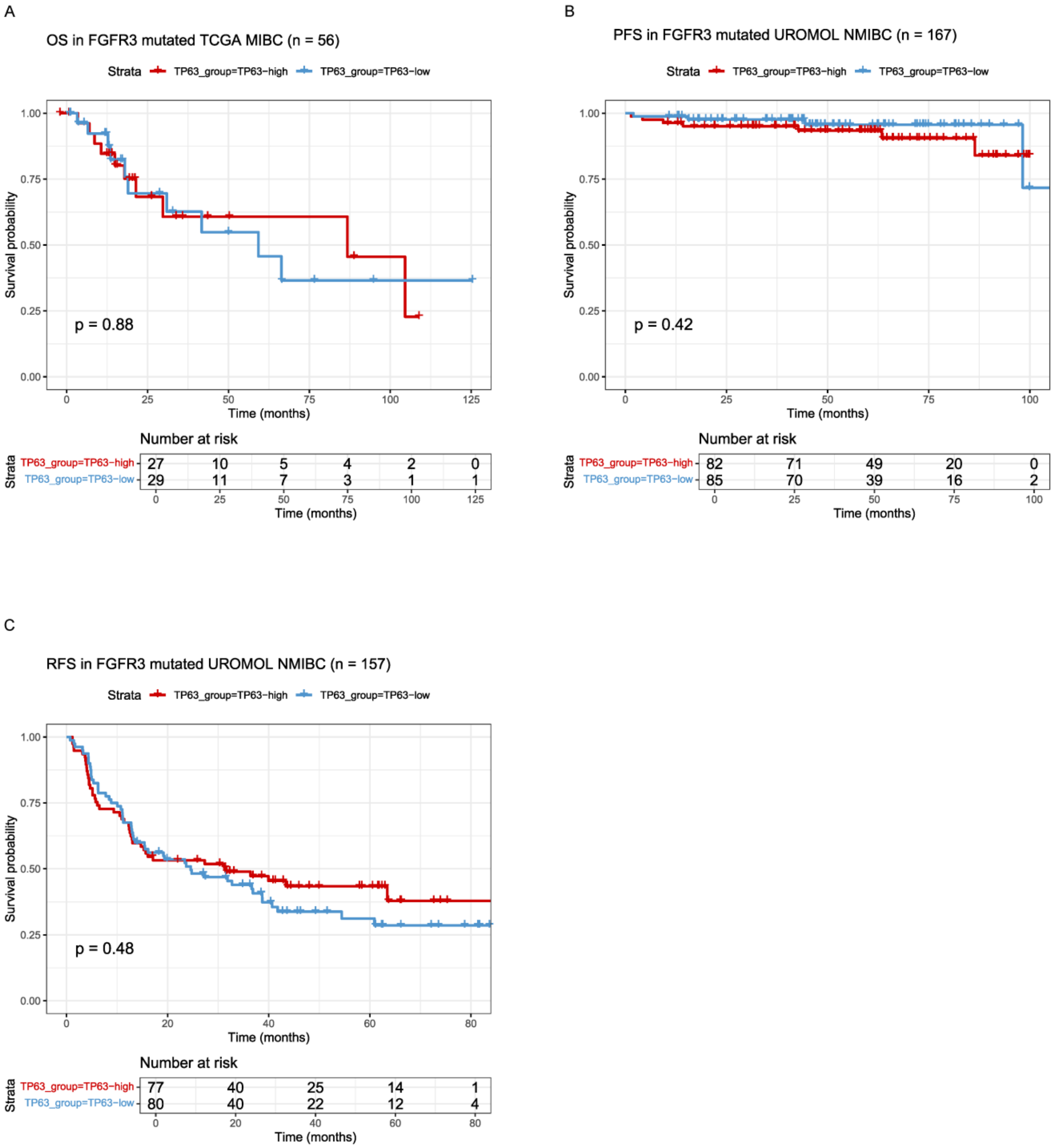

Figure S3: Lack of association between TP63 activity and clinical outcome in *FGFR3*-mutated tumors.

A) Overall survival (OS) of *FGFR3*-mutated MIBC patients from the TCGA cohort stratified by high versus low TP63 regulon activity (cut by median). **B-C)** Progression-free survival (PFS) (B) and recurrence-free survival (RFS) of *FGFR3*-mutated NMIBC patients from the UROMOL cohort stratified by TP63 activity.

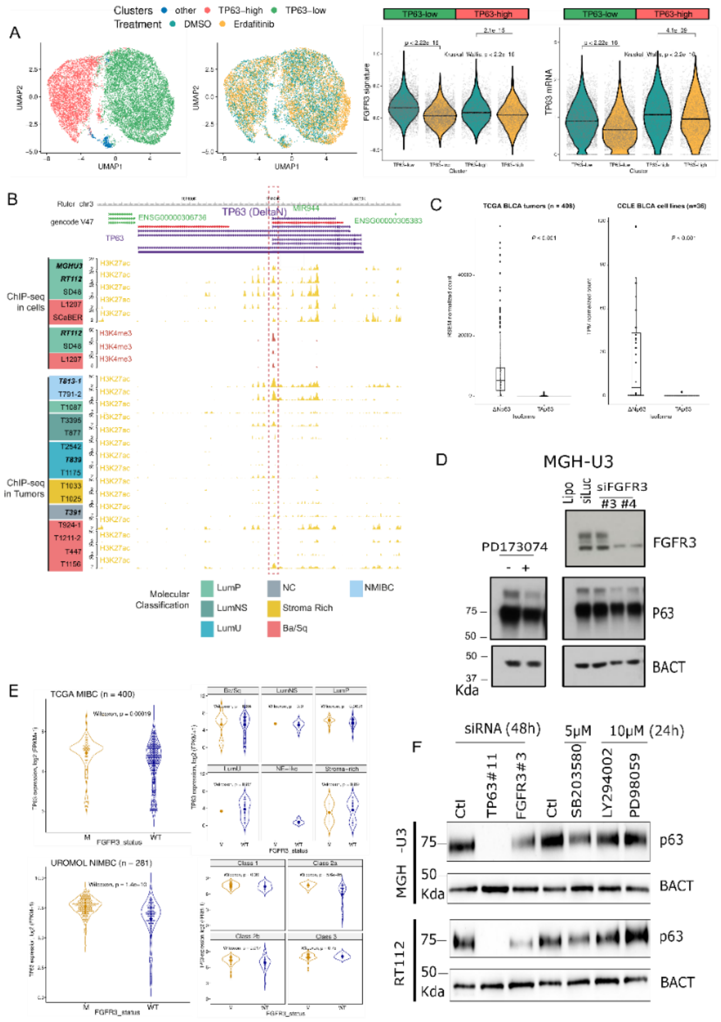

Figure S4: FGFR3 regulates TP63 expression predominantly through the ΔNp63 isoform

A) Single-cell RNA-seq analysis of MGH-U3 cells treated with erdafitinib showing reduced TP63 expression and FGFR3 pathway activation score across both TP63-high and TP63-low populations identified figure 4D following FGFR inhibition. Comparisons between clusters were performed using the Kruskal–Wallis test. Comparisons between TP63-low and TP63-high clusters were performed using the Wilcoxon rank-sum test. **B)** ChIP-seq profiles of active chromatin marks (H3K27ac and H3K4me3) at the TP63 locus in bladder cancer cell lines and tumors, showing selective activation of ΔNp63 promoter and enhancer regions. **C)** RNA-seq–based isoform of ΔNp63 over TAp63 in bladder cancer cell lines from CCLE (n=36) and human tumors from the TCGA cohort **(D)** Western blot analysis confirming predominant expression of short p63 isoform but efficient knockdown of both p63 isoforms following FGFR3 inhibition during 40h with 500 nM PD173074 pan-FGF-inhibitor or 48 hours after FGFR3 siRNA treatment in MGH-U3 FGFR3-dependent cells. **E)** TP63 mRNA expression in *FGFR3*-mutated versus *FGFR3*–wild-type NMIBC and MIBC tumors from the UROMOL and TCGA cohorts, respectively. Statistical comparisons were performed using Wilcoxon rank-sum test. **F)** Western blot analysis of TP63 expression in FGFR3-dependent bladder cancer cell lines, RT112 and MGHU-3 treated 48h with p38 (SB203580) or PI3K (LY294002) inhibitors. Samples were from (9) and with already demonstrated potent p-p38 and p-AKT inhibition after treatment associated with a decrease in MYC expression.

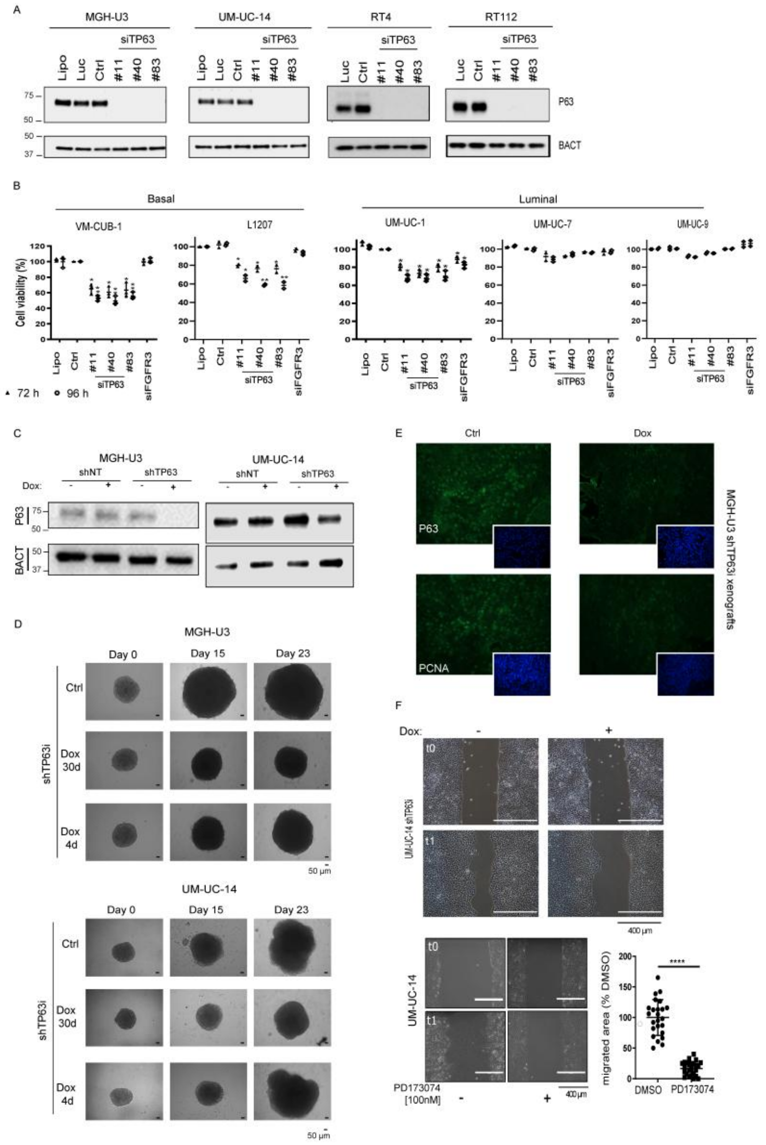

Figure S5: Validation of TP63 knockdown efficiency and functional assays.

A) FGFR3-dependent cells were transfected with three different siRNAs targeting *TP63* (siTP63 #11, #40, #83). Forty-eight hours after transfection, the cell lysates were recovered and analyzed by immunoblotting with antibodies against p63. Actin (BACT) was used as a loading control. **B)** Effect of TP63 knockdown on FGFR3-independent cell viability assessed by cellTitleGlo 72h or 96h post transfection**.** C**)** MGH-U3 and UM-UC-14 cells stably expressing doxycycline (Dox)-inducible shRNA directed against *TP63* (shTP63i) were treated with or without Dox, and the efficiency of knockdown was corroborated by western blotting of p63. Actin (BACT) was used as a loading control. **D)** Representative microscopy images of MGH-U3 shTP63i#4 and UM-UC-14 shTP63i#4 cells treated with or without Dox for a long (30 days; 30 days) or short (4 days; 4 days) period. The scale bar represents 50 µm. **E)** Representative immunofluorescence images of p63-stained cells in tumors from xenografted mice generated from MGH-U3 shTP63i#4 cells and treated with or without Dox for 30 days. **F)** Wound healing assay to measure cell migration. Top panel. UM-UC-14 shTP63i#4 cells after doxycycline (Dox)-induced knockdown of *TP63*. Bottom panel. UM-UC-14 cells were treated with the pan-FGFR inhibitor PD173074 [100 nM] for 48 hr. Representative images depicting the scratch (wound) at 0 (t0) and 24 or 48 hours (t1) post scratching. The scale bar is equivalent to 100 µm (left). Quantification of migration and statistical comparison using an unpaired 2-tailed t test (right).

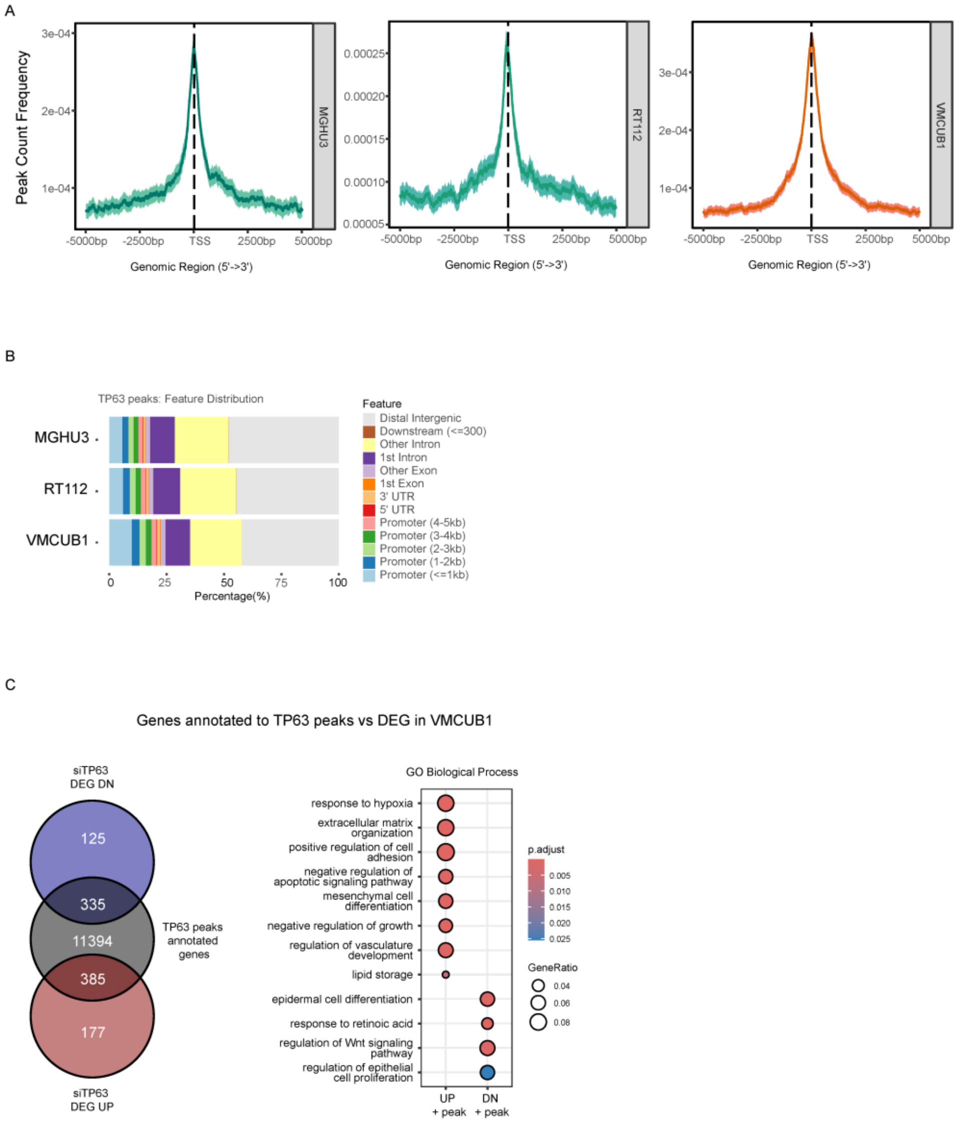

Figure S6: p63 ChIP-seq peak annotation and integration with transcriptomic analysis after TP63 knockdown.

A) Distribution of p63 ChIP-seq peak density relative to transcription start sites (TSS ±5 kb) in MGH-U3, RT112, and VMCUB1 bladder cancer cell lines. **B)** Genomic annotation of p63 ChIP-seq peaks across the three cell lines. Peaks are classified by genomic features, including promoter, intronic, exonic, and intergenic regions. **C)** Venn diagram comparing genes associated to a p63 peak, and genes differentially regulated upon siRNA-mediated knockdown of *TP63* in VMCUB1. The intersections are considered as p63 direct targets (left). Gene Ontology enrichment analysis of direct p63 target genes (right) using GO Biological Processes Database.

	Strand	Sequence 5'-3'
TP63 #11 *ref (4392420 s16411)*	sense	GGAUGAAGAUAGCAUCAGA
	anti-sense	UCUGAUGCUAUCUUCAUCC
TP63 #40 *ref (4392420 s229400)*	sense	GAACCGCCGUCCAAUUUU
	anti-sense	UAAAAUUGGACGGCGGUU
TP63 #83 *ref (4392420 s531583)*	sense	UGAUGAACUGUUAUACUU
	anti-sense	UAAGUAUAACAGUUCAUCA
FGFR3 #4 *ref (4392420 s5168)*	sense	CCUGCGUCGUGGAGAACAATT
	anti-sense	UUGUUCUCCACGACGCAGGTG

Gene	Strand	Sequence 5' - 3'	Roche T*aq*man probe
ΔNp63	sense	GGTTGGCAAAATCCTGGAG	No. 56
	antisense	GGTTCGTGTACTGTGGCTCA	
18 s rRNA	Sense	GGAGAGGGAGCCTGAGAAAC	No. 8
	Antisense	TCGGGAGTGGGTAATTTGC	

Table S4 : list of primers and probes used for RT-qPCR
