# Supplementary material for: FGFR3-driven gene regulatory network analysis reveals a protumoral role for p63 in luminal bladder tumors
Source: J Clin Invest. 2026 Aug 3;136(15):e193280. doi: 10.1172/JCI193280 (PMC13430010; doi:10.1172/JCI193280)
Supplement: Unedited blot and gel images [file jci-136-193280-s008.pdf]

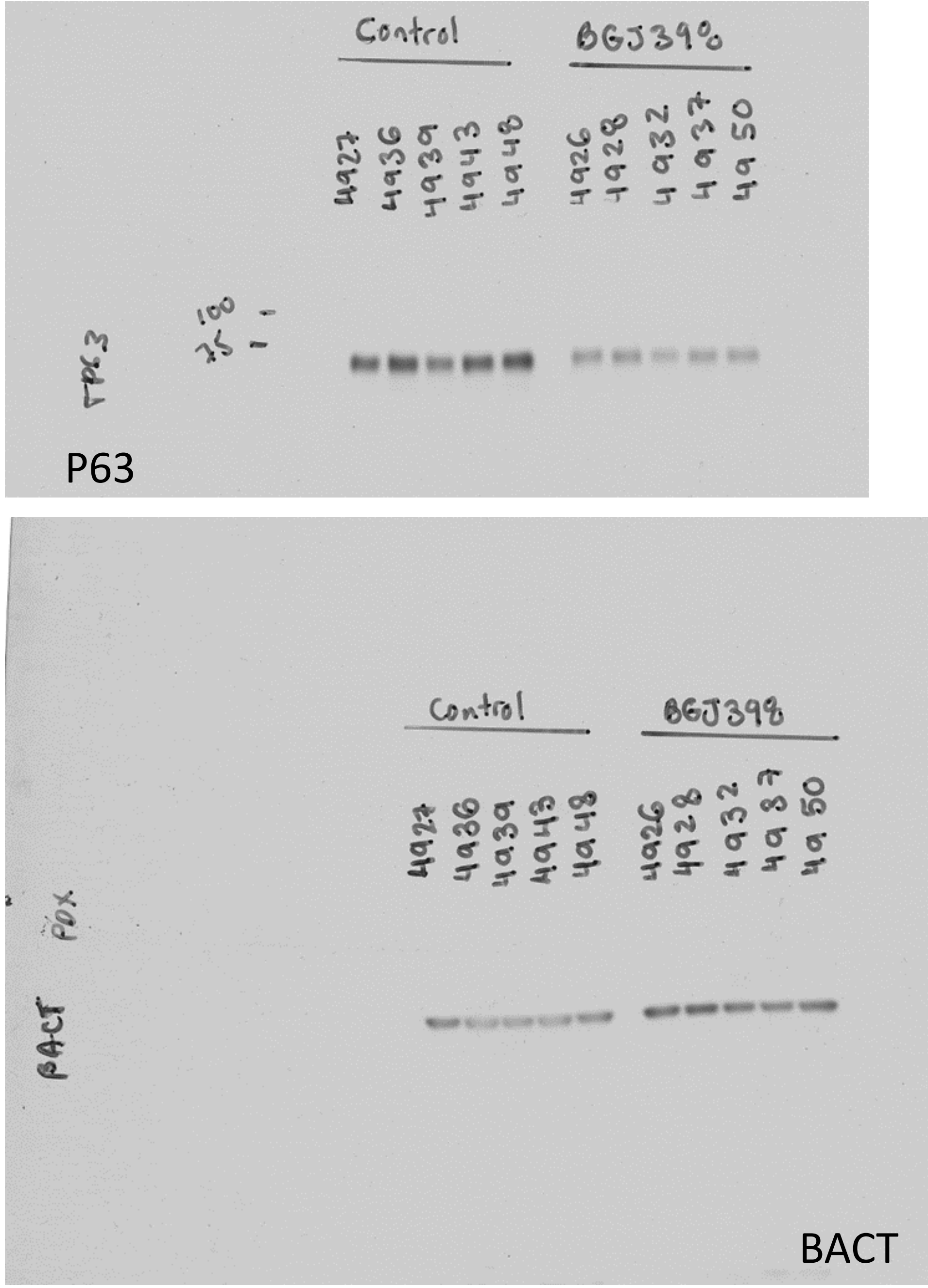

unedited western blot for Figure 4C- right panel

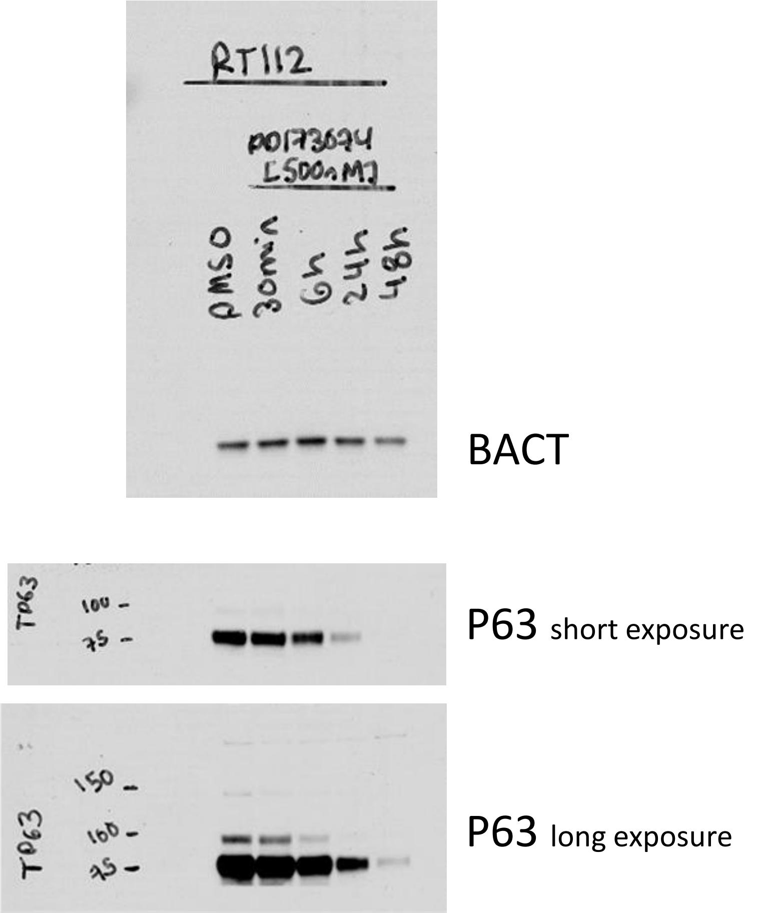

unedited western blot for Figure 4D

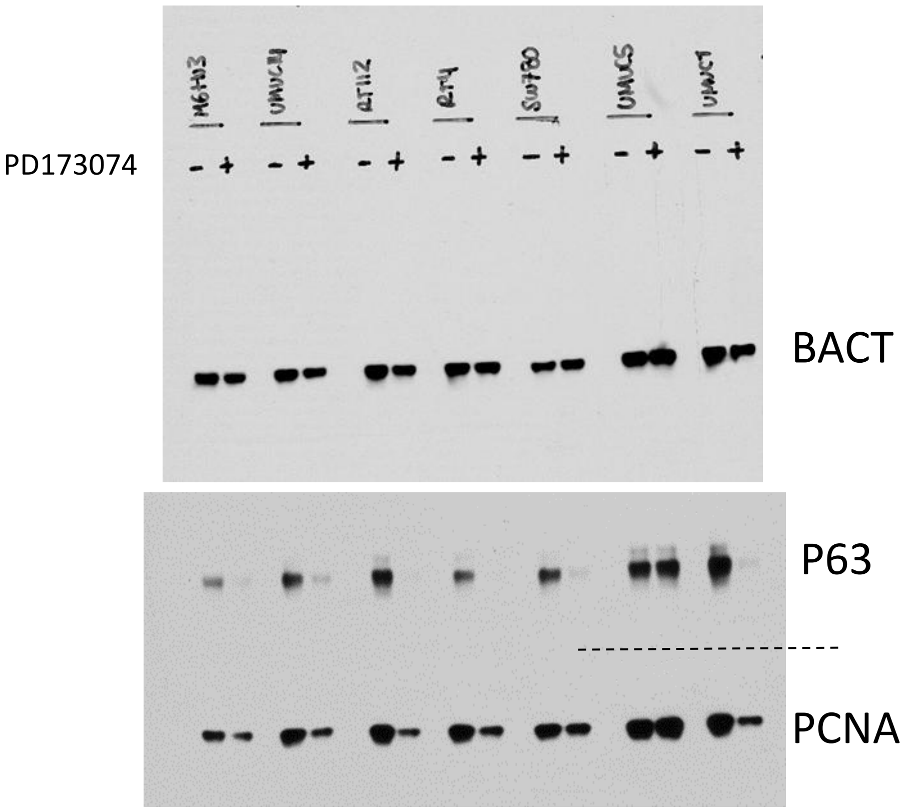

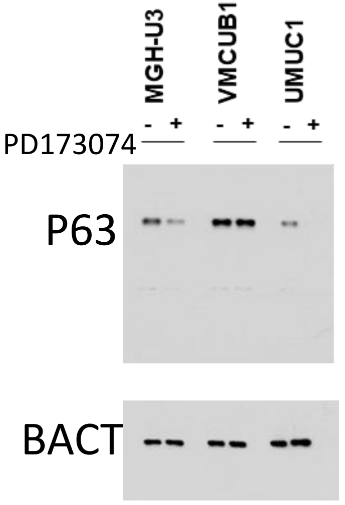

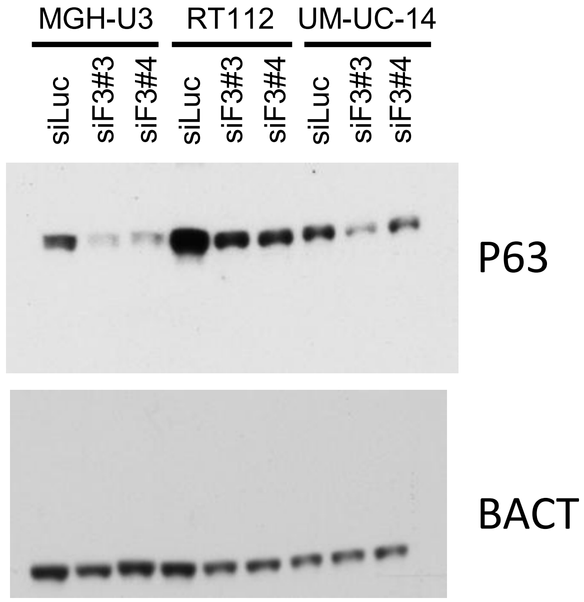

unedited western blot for Figure 4C- left panel

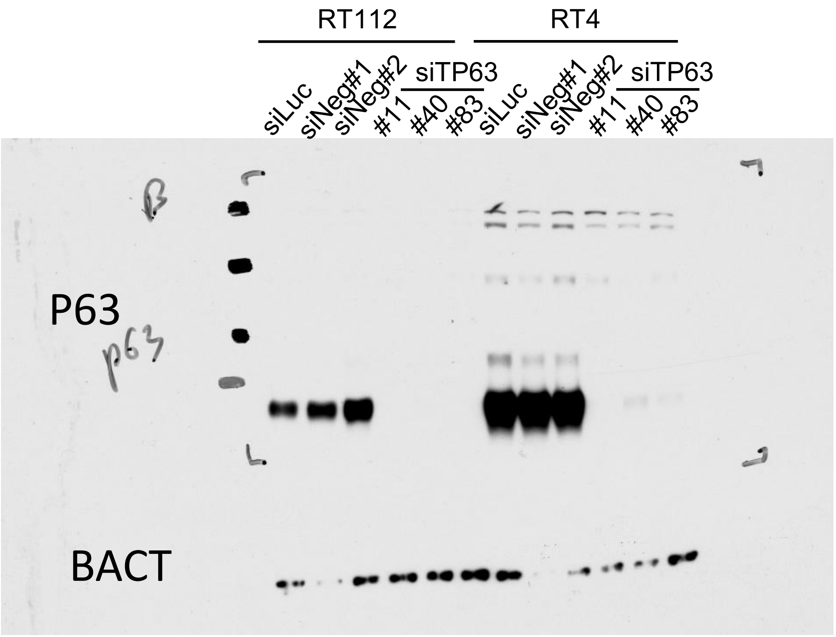

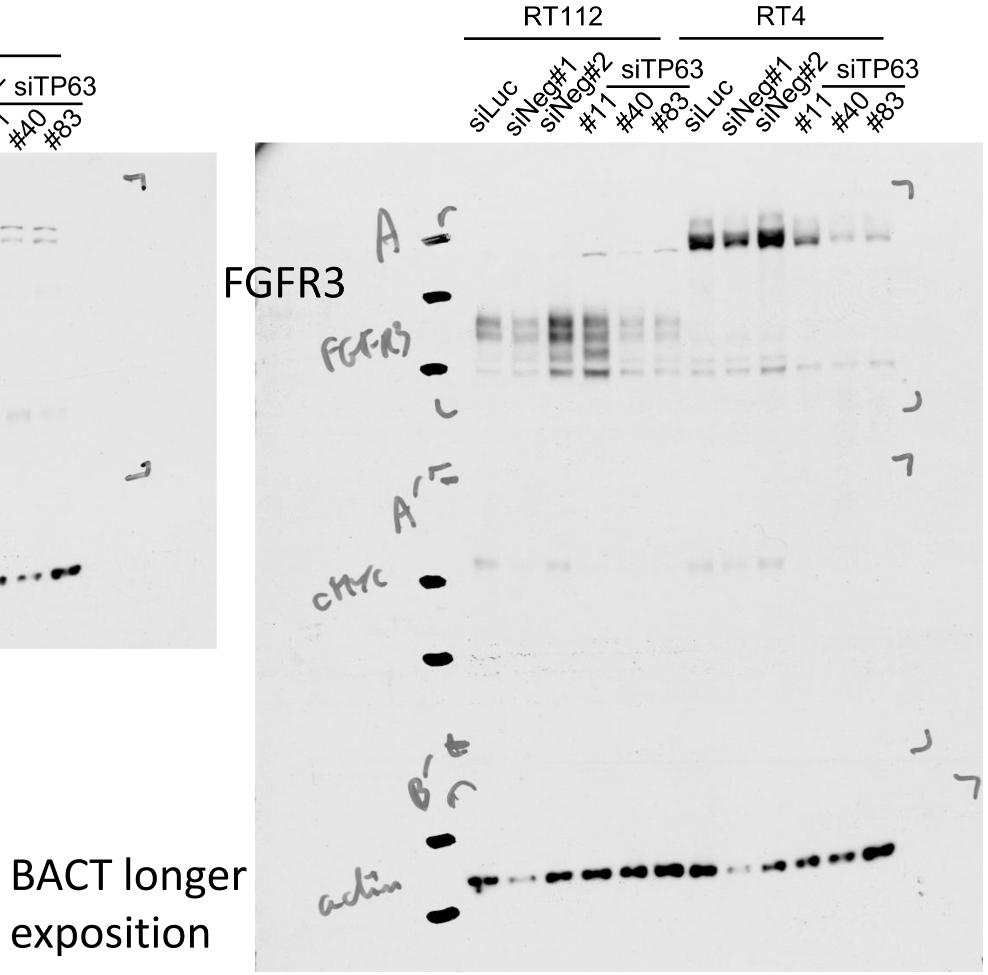

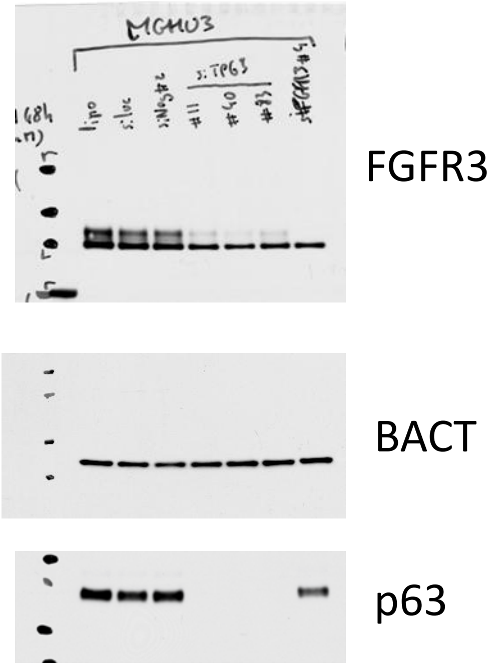

unedited western blot for Figure 6E

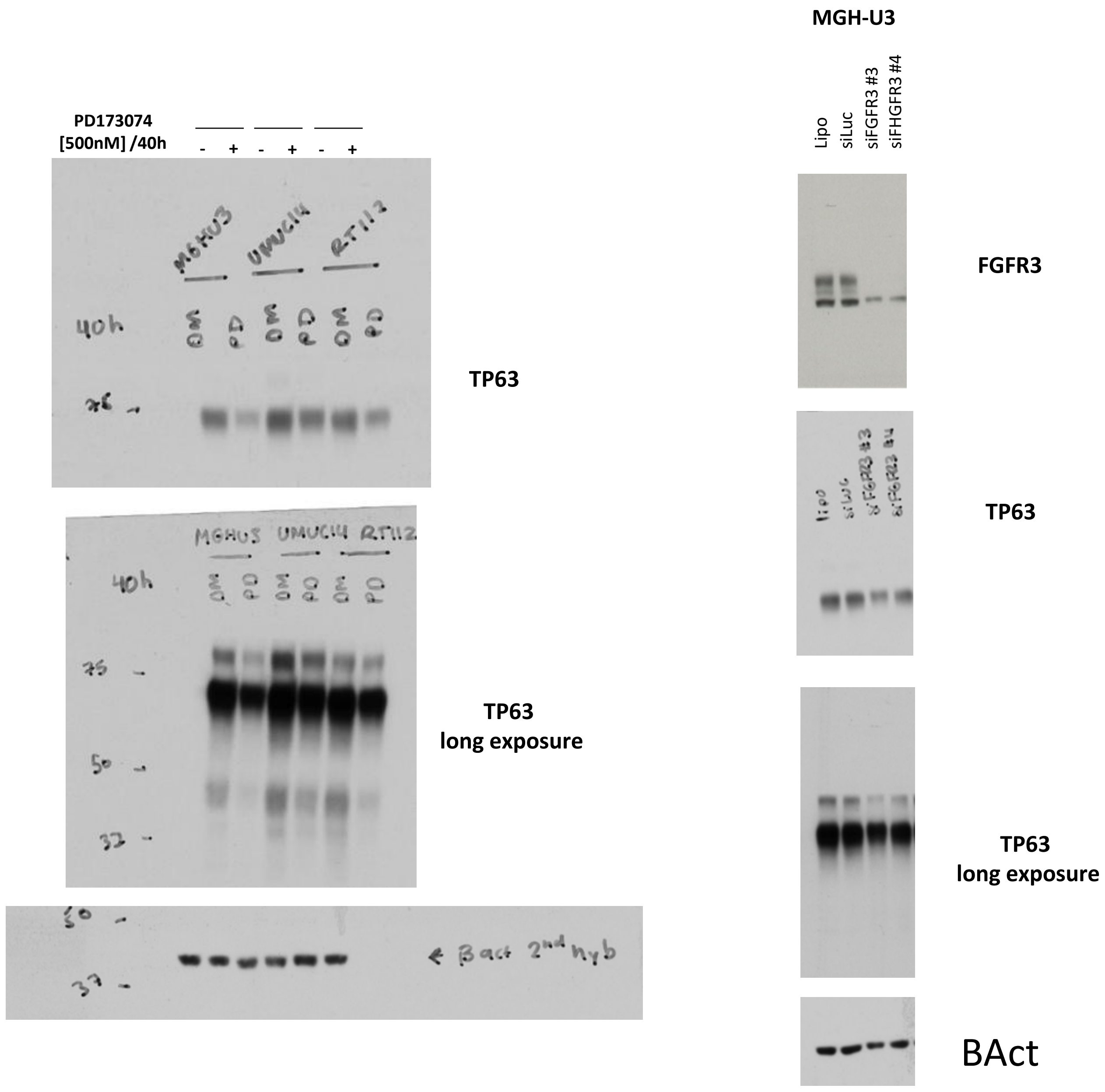

unedited western blot for Figure S4D

MGH-U3

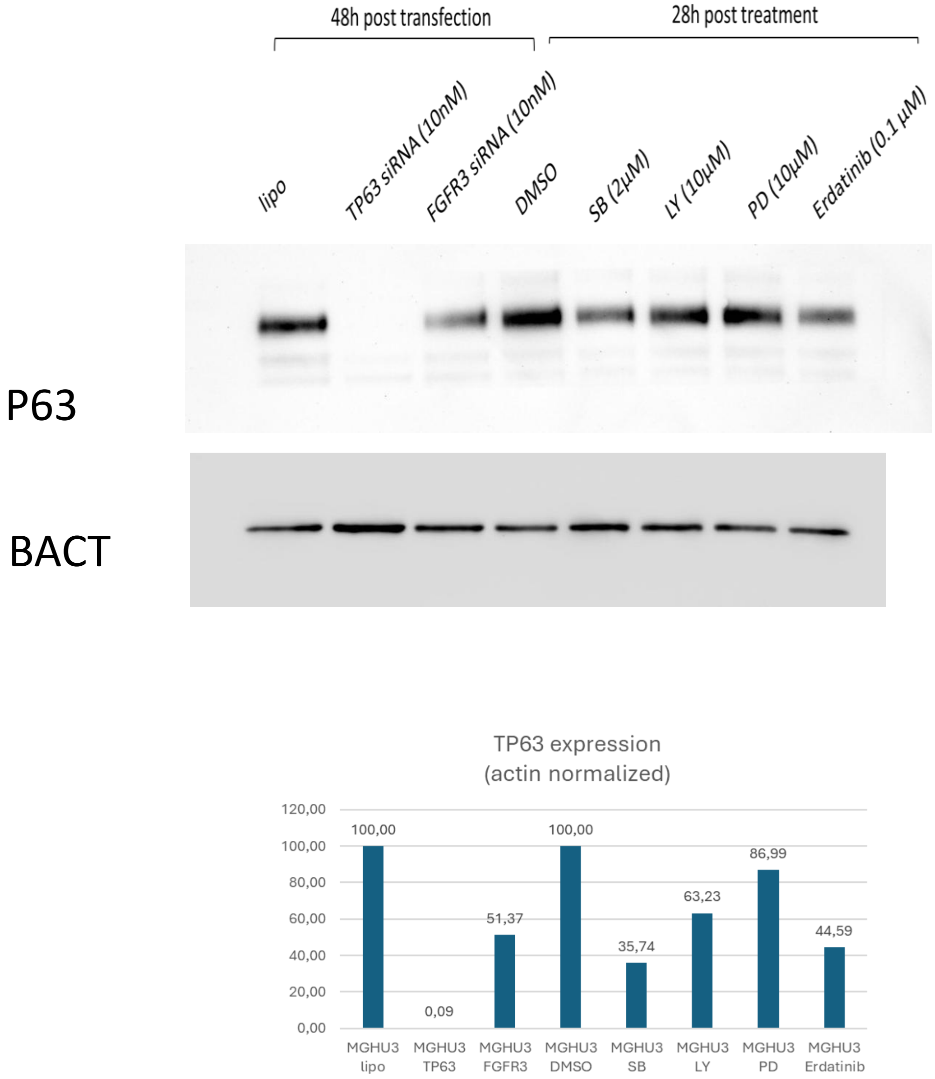

RT112

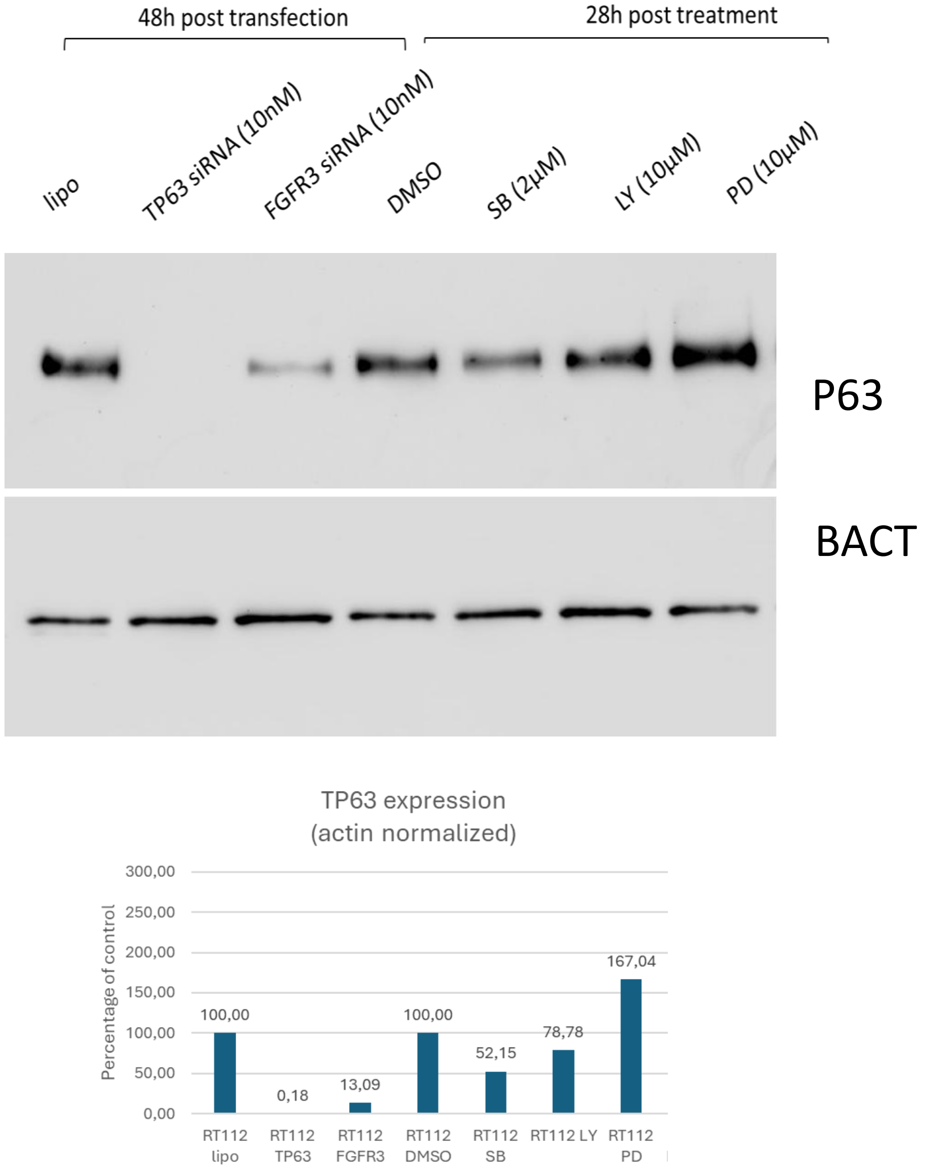

unedited western blot for Figure S4F

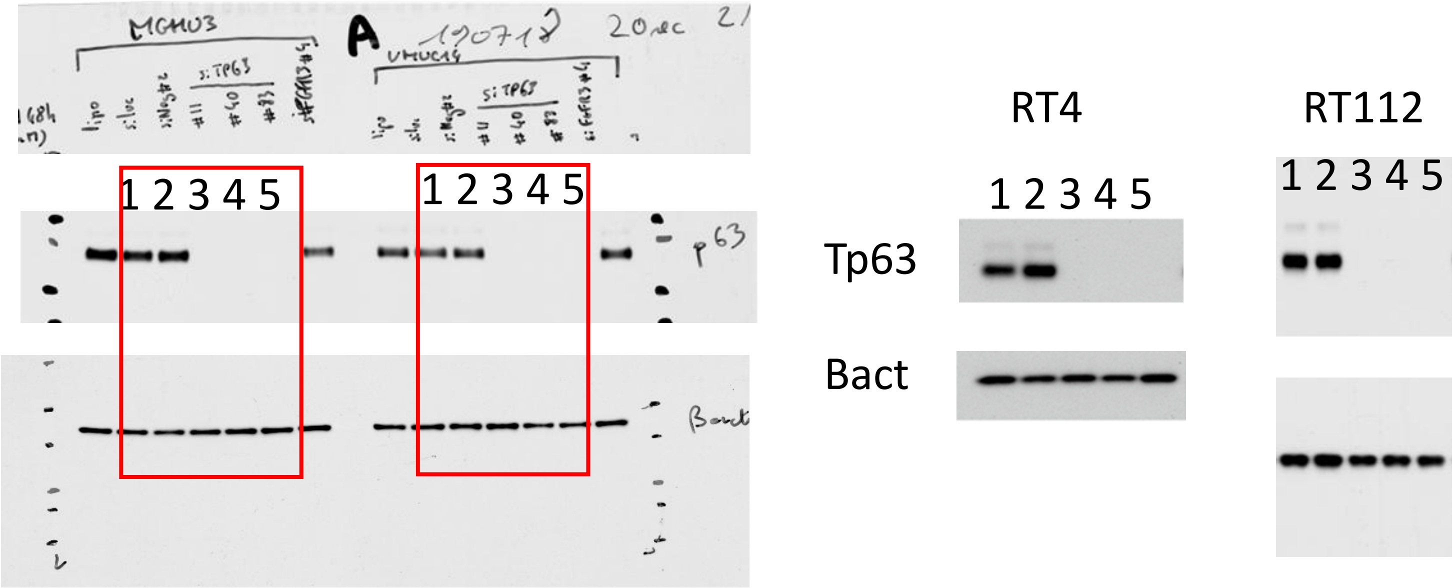

1-SiLuc
2-SiNeg#2
3-SiTP63#11
4-SiTP63#40
5-SiTP63#83

unedited western blot for Figure S5A

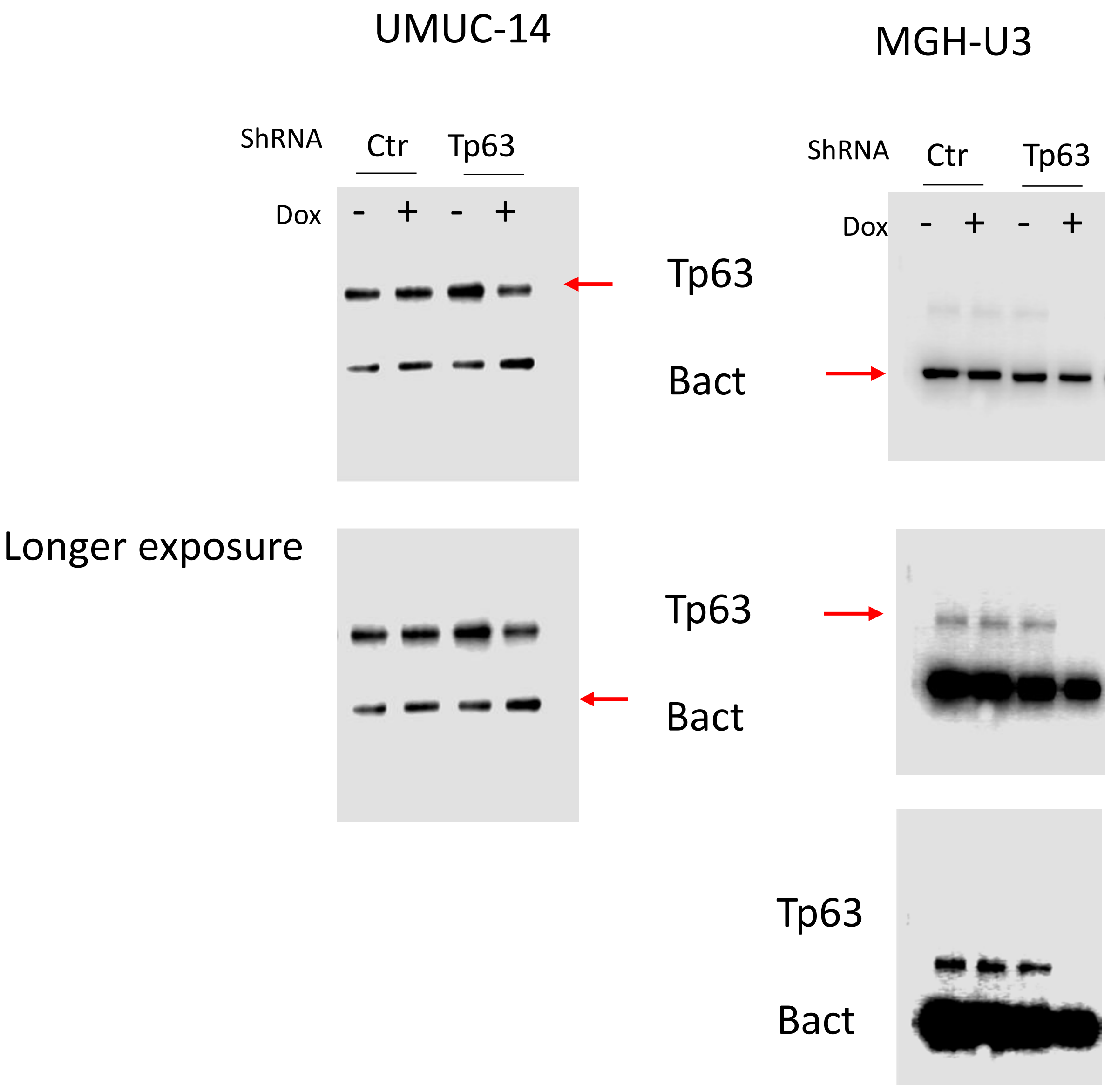

unedited western blot for Figure S5C
